# Bioinformatics-Driven Systematic Molecular Typing and Rapid qPCR Detection of *Escherichia coli* Phages: Preliminary Validation with Isolates from Cattle Farms in Xinjiang

**DOI:** 10.3390/pathogens15010121

**Published:** 2026-01-22

**Authors:** Xinyu Dang, Xiaoguang Cao, Li Li, Lin Yang, Lei Zhao, Jinliang Sheng, Xin Zheng, Chunyan Zhai, Jia Song, Wenhui Wu, Yongjie Wang, Shilei Zhang

**Affiliations:** 1 College of Animal Science and Technology, Shihezi University, Shihezi 832003, China; 2 Central Blood Station of Shihezi City, 8th Division, Xinjiang Production and Construction Corps, Shihezi 832000, China; 3 Central Blood Station, Xinjiang Production and Construction Corps, Urumqi 830002, China; 4 Department of Animal Sciences, College of Agriculture and Environmental Sciences, North Carolina Agricultural and Technical University, Greensboro, NC 27401, USA

**Keywords:** *Escherichia coli* phage, phage classification and identification, PCR, qPCR, environmental surveillance

## Abstract

This study aimed to classify *Escherichia coli* phages using bioinformatics analysis systematically and to establish corresponding PCR and qPCR detection methods for rapid molecular typing and identification. Based on 419 complete *E. coli* phage genomes available in NCBI, phylogenetic and pan-genomic analyses were conducted to classify the phages at the family, subfamily, and genus levels and to identify highly conserved core genes. Specific primers targeting these core genes were designed, and their specificity, sensitivity, and reproducibility were verified using conventional PCR and dye-based qPCR. A total of 357 phages were successfully classified, encompassing 10 families, 20 subfamilies, and 67 genera. Pan-genomic analysis identified type-specific core genes within 16 taxa, including *Ackermannviridae* and *Demerecviridae*, for which 16 pairs of primers were designed. Validation using bacteriophages isolated from Xinjiang cattle farms showed distinct single PCR bands with high specificity, and the qPCR assay achieved a sensitivity of up to 10^−5^ µg/µL. This study established an efficient and broad-spectrum molecular typing and detection method for *E. coli* phages, providing a powerful preliminary screening tool for phage selection.

## 1. Introduction

With the escalating problem of antibiotic resistance, the prevalence of multidrug-resistant bacterial strains has become a significant threat to public health security [1]. Phages, viruses that specifically infect and lyse bacteria, have attracted increasing attention due to their unique mechanisms of action and high host specificity. They hold great potential for addressing bacterial resistance, biological control, and contamination monitoring in industrial fermentation processes. Whether for eliminating mild, often undetectable phage contamination in the fermentation industry or for isolating virulent phages used in phage therapy, rapid and accurate screening and identification are crucial. Traditional isolation and identification methods, such as the double agar overlay assay, are labor-intensive, time-consuming, and low-throughput, limiting the efficient use of phages. Although molecular biology techniques such as PCR [2,3] and sequencing [4] have been applied to phage detection, current approaches still have notable limitations. Most existing PCR-based methods target virulent phages infecting specific bacterial species, such as *Klebsiella pneumoniae* or *Staphylococcus aureus* [5], but are incapable of detecting temperate phages, which represent major hidden threats to industrial contamination. In addition, PCR assays targeting single phage genes, such as those for *Erwinia* phages [6], have a narrow detection range and fail to meet the need for large-scale, systematic screening across diverse phage communities. The rapid advancement of bioinformatics and the continuous expansion of genomic databases have provided new opportunities to address these challenges [4,5,6]. Bioinformatics tools enable comprehensive analysis of phage genomic features and facilitate the identification of highly conserved core genes at the family and genus levels. These core genes can serve as molecular markers for developing broad-spectrum detection systems capable of distinguishing taxonomic units while simultaneously covering both virulent and temperate phages.

In this study, by integrating bioinformatics analysis, pan-genome profiling, and molecular detection technologies, we used *Escherichia coli* phages—a diverse and representative group—as a model to systematically identify conserved molecular markers and establish a sensitive and specific PCR and qPCR detection system. This work provides an efficient technical platform to support subsequent phage research and applications.

## 2. Materials and Methods

### 2.1. Genomic Data Collection and Comprehensive Bioinformatic Analysis

**Figure 1:**
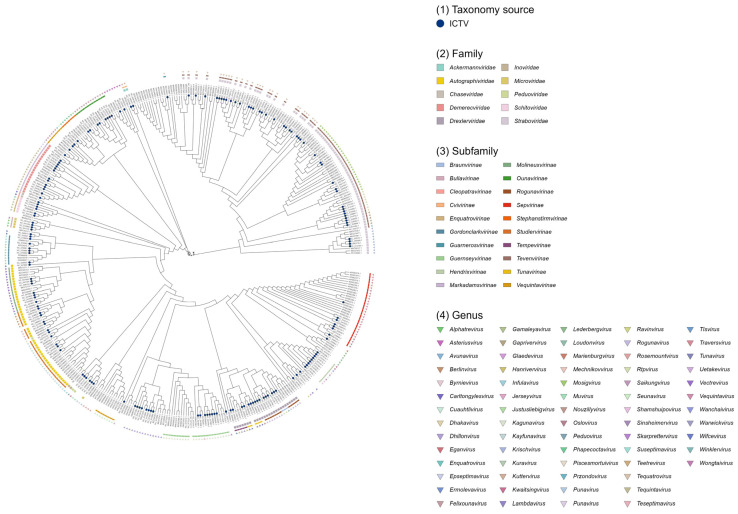
Phylogenetic tree of 419 *Escherichia coli* phages.

A total of 419 complete *Escherichia coli* phage genome datasets (Figure 1) were retrieved and downloaded from the NCBI RefSeq or GenBank databases using Batch Entrez (https://www.ncbi.nlm.nih.gov/sites/batchentrez, accessed on 16 October 2024). Gene annotation was performed using Prokka v1.14.6 [7], supplemented with the pVOG (2017) [8], PHROG v3 [9], and Caudovirales (https://millardlab.org/, accessed on 24 December 2024) databases. Phylogenetic trees based on pairwise genomic distances among phages were inferred using the locally installed ViPTreeGen v1.1.2 [10]. For 177 genomes, official taxonomic classification was obtained from the ICTV Master Species List 2023.v4 (https://ictv.global/taxonomy, accessed on 24 December 2024). Phage genomes without ICTV-assigned taxonomy were classified to the family and genus levels using vConTACT 2 v0.9.22 [11] with default parameters, based on the “Prokaryotic Viral RefSeq 211-Merged” database [4]. All phylogenetic trees were midpoint-rooted and visualized using RStudio v2022.02.3. To predict virulent or temperate lifestyles of the 419 *E. coli* phages, BACPHLIP v0.3.4 [12] was applied with default settings.

### 2.2. Bioinformatic Strategies for Pan-Genome Reconstruction and Core Gene Identification

The GFF3 annotation files generated by Prokka were used to construct the pan-genome. Pan-genome analysis was performed using Roary v3.11.2 [13] with an amino acid identity threshold of 0.95. The results were visualized using the roary_plots.py script (Roary/contrib/roary_plots/roary_plots.py at master · sanger-pathogens/Roary). In addition, Pirate v1.0.4 [14] was employed for supplementary pan-genome analysis, with amino acid identity thresholds set at 30%, 40%, 50%, 60%, 70%, 80%, 90%, and 95%. FASTA-formatted sequences were processed using SeqKit v2.9.0 [15]. Core genes were identified based on the comprehensive analysis of the pan-genome results.

### 2.3. Primer Design Based on Conserved Core Gene Sequences

Genes selected for each operational taxonomic unit (OTU) typing system were used to design primers. Orthologous genes with >60% sequence homology and an average length exceeding 400 bp were retained for further analysis. Pairwise alignments of orthologous gene families were performed using MAFFT v7.525 [16] with default parameters. Primer design was conducted using MEGA v11 and Oligo v6.31, while primer pair specificity was evaluated using the NCBI Primer-BLAST online tool (https://www.ncbi.nlm.nih.gov/tools/primer-blast/, accessed on 24 December 2024) [17].

### 2.4. PCR Verification of Primer Specificity

Four *Escherichia coli* phage strains were used to verify the specificity of the designed primers by PCR. Among them, two clinical isolates obtained from cattle farms in Xinjiang were previously sequenced and identified as vB_EcoP_SP7 (MT682707.1) and EP335 (NC070979.1), while the standard phages T4 and T7 were purchased from Biotechnology Co., Ltd. (Hangzhou, China). Phage DNA was extracted and tenfold serially diluted for use as templates. The PCR reaction mixture (20 μL total volume) contained 0.5 μL each of forward and reverse primers, 2 μL of DNA template, 10 μL of 2× Taq Mix, and 7 μL of ddH_2_O. The thermal cycling program consisted of an initial denaturation at 94 °C for 5 min, followed by 30 cycles of 94 °C for 30 s (denaturation), annealing at the appropriate temperature for 30 s, and extension at 72 °C for 90 s, with a final extension at 72 °C for 5 min. Gradient-dilution PCR assays were performed using the phage DNA templates to assess amplification efficiency and specificity.

### 2.5. Validation of Primer Sensitivity and Specificity via qPCR

The sensitivity of the designed primers was verified using *Escherichia coli* phages available in the laboratory. Phage DNA was extracted and serially diluted tenfold for use as templates. The qPCR reactions were performed in a total volume of 20 μL, containing 0.5 μL each of forward and reverse primers, 2 μL DNA template, 10 μL 2× SYBR Green Taq Mix (Thermo Fisher Scientific, Waltham, MA, USA), and 7 μL ddH_2_O. The amplification program was as follows: Stage 1: initial denaturation at 95 °C for 30 s; Stage 2: 40 cycles of denaturation at 95 °C for 10 s, annealing and extension at 60 °C for 30 s; Stage 3: melting curve analysis consisting of 95 °C for 15 s, 60 °C for 60 s, gradual heating from 60 °C to 95 °C, and a final step at 95 °C for 15 s. Multiplex PCR verification was also performed using mixed primer sets.

## 3. Results

### 3.1. Phylogenetic Analysis of Escherichia coli Phages

Based on the annotated FAA files generated by Prokka (Attachment S1: NCBI *Escherichia coli* phage FASTA), the taxonomic predictions obtained from vConTACT 2 (Attachment S2: genome_by_genome_overview), and the existing ICTV classifications, phylogenetic trees of 419 *E. coli* phages were constructed using the ViPTreeGen analysis results (Attachment S3: all.bionj.asc.newick). The resulting trees were visualized in RStudio (Figure 1). The analysis showed that 177 *E. coli* phages (approximately 42.2%) had been officially classified by ICTV, while 180 phages were taxonomically assigned by vConTACT 2 predictions. The remaining 62 phages lacked family- or genus-level classification from either the ICTV or vConTACT 2. Among the 357 phages with established taxonomy, they were distributed across 10 families, 20 subfamilies, and 67 genera. Lifestyle prediction of the phages using BACPHLIP (Attachment S4: bacphlip_summary) indicated that 298 phages were virulent and 121 were temperate (Table 1).

### 3.2. Pan-Genome Analysis and Core Gene Prediction

**Figure 2:**
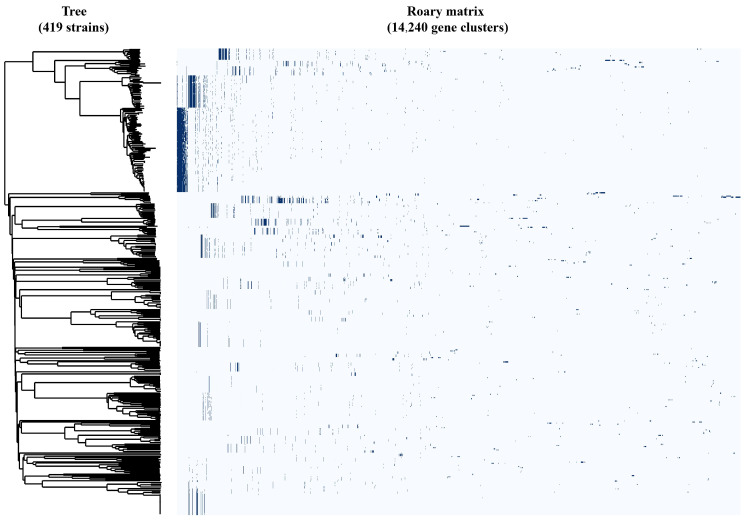
Identification of core genes in *Escherichia coli* phages based on pan-genome analysis.

The GFF3 annotation files generated by Prokka (Attachment S5: Prokka_GFF) were used as input for pan-genome analysis with Roary and Pirate. The results revealed that among the 419 *Escherichia coli* phages, core genes were identified at multiple taxonomic levels: at the family level—*Ackermannviridae*, *Chaseviridae*, *Demerecviridae*, *Inoviridae*, and *Straboviridae*; at the subfamily level—*Molineuxvirinae*, *Studiervirinae*, *Tunavirinae*, *Bullavirinae*, *Gordonclarkvirinae*, *Guarnerosvirinae*, *Hendrixvirinae*, *Ounavirinae*, *Sepvirinae*, and *Stephanstirmvirinae*; and at the genus level—Dhillonvirus (Figure 2, Table 2).

Specifically, the core genes of *Ackermannviridae*, *Molineuxvirinae*, *Chaseviridae*, *Demerecviridae*, *Tunavirinae*, *Guarnerosvirinae*, *Hendrixvirinae*, *Ounavirinae*, *Sepvirinae*, *Stephanstirmvirinae*, and *Dhillonvirus* were identified through Roary analysis (Attachment S6: Pan-genome analysis using Roary software) with amino acid identity greater than 95%. The remaining taxa were analyzed using Pirate results (Attachment S7: Pan-genome analysis using Pirate software).

### 3.3. Batch Primer Design Based on Conserved Core Gene Sequences

Primers were designed based on the tagged genes identified from the pan-genome analysis. Among these, DNA ligase (*Molineuxvirinae*), dUTPase (*Chaseviridae*), polA (*Demerecviridae*), putative DNA repair helicase RadD (*Tunavirinae*), grcA (*Straboviridae*), and lysozyme RrrD (*Ounavirinae*) were annotated as known protein-coding genes, while the remaining targets corresponded to hypothetical protein genes (Table 3). Multiple sequence alignments of the tagged genes were performed using MAFFT (Attachment S8: Results of core gene comparison analysis using MAFFT software), and primers were designed from the conserved regions obtained. Primer specificity was verified using the NCBI Primer-BLAST tool (Attachment S9: Primer-BLAST verification results of primer specificity).

### 3.4. PCR Verification of Primer Specificity and Sensitivity

**Figure 3:**
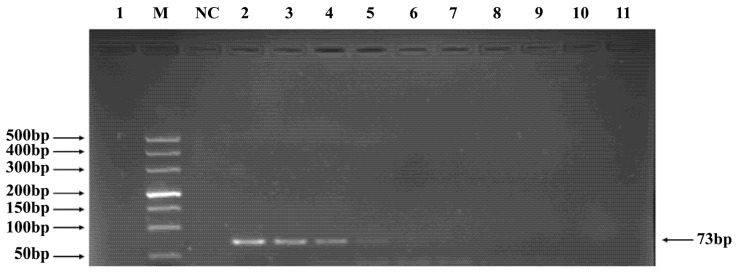
PCR verification results for *Studiervirinae* phage primers. Note: M, DNA marker; NC, negative control; 1, cross-reaction control; 2–11, template concentrations ranging from 4.54 μg/μL × 10^−0^~10^−9^.

**Figure 4:**
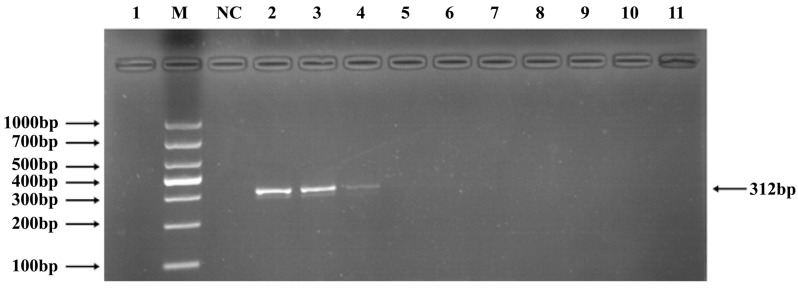
PCR verification results for *Straboviridae* phage primers. Note: M, DNA marker; NC, negative control; 1, cross-reaction control; 2–11, template concentrations ranging from 2.08 μg/μL × 10^−1^~10^−10^.

**Figure 5:**
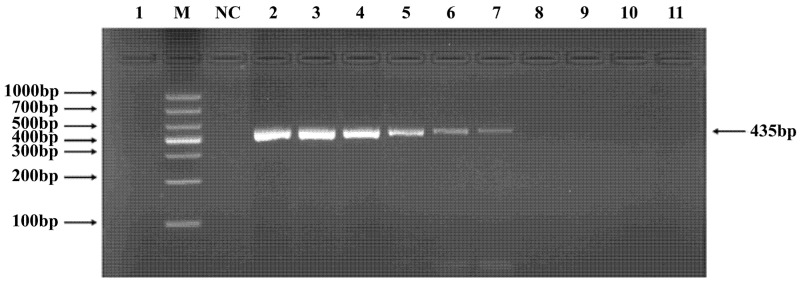
PCR verification results for *Gordonclarkvirinae* phage primers. Note: M, DNA marker; NC, negative control; 1, cross-reaction control; 2–11, template concentrations ranging from 1.20 μg/μL × 10^2^~10^−7^.

**Figure 6:**
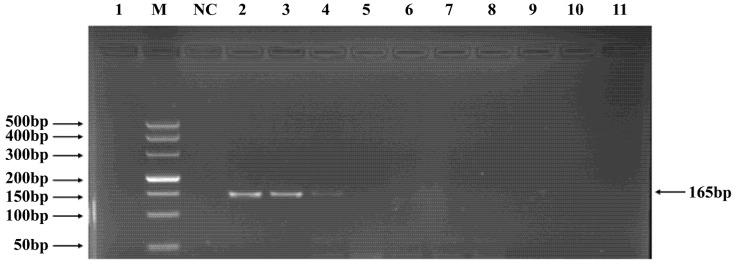
PCR verification results for *Tunavirinae* phage primers. Note: M, DNA marker; NC, negative control; 1, cross-reaction control; 2–11, template concentrations ranging from 2.96 μg/μL × 10^−1^~10^−10^.

Phages belonging to four taxonomic groups—*Studiervirinae*, *Straboviridae*, *Gordonclarkvirinae*, and *Tunavirinae*—available in the laboratory were used to verify the specificity of the designed primers by PCR. Phage DNA was extracted and serially diluted tenfold to serve as templates. Agarose gel electrophoresis of the PCR products showed clear and distinct bands, confirming primer specificity. The minimum detectable DNA concentrations for the primer sets corresponding to *Studiervirinae*, *Straboviridae*, *Gordonclarkvirinae*, and *Tunavirinae* were 4.54 × 10^−3^ μg/μL, 2.08 × 10^−3^ μg/μL, 1.20 × 10^−3^ μg/μL, and 2.96 × 10^−3^ μg/μL, respectively (Figure 3, Figure 4, Figure 5 and Figure 6).

### 3.5. qPCR Verification of Primer Sensitivity

**Figure 7:**
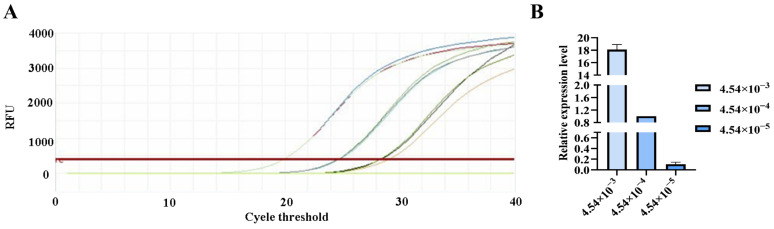
qPCR amplification curves for *Studiervirinae* primers. (**A**) Amplification curves for varying concentrations of phage DNA templates; (**B**) Relative quantification of the templates (normalized to the median concentration group).

**Figure 8:**
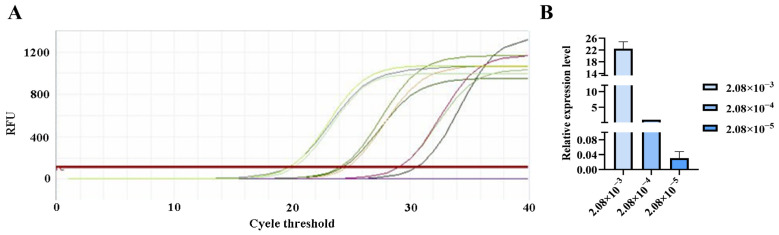
qPCR amplification curves for *Straboviridae* primers. (**A**) Amplification curves for varying concentrations of phage DNA templates; (**B**) Relative quantification of the templates (normalized to the median concentration group).

**Figure 9:**
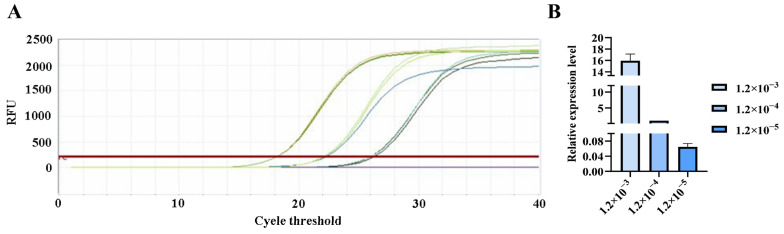
qPCR amplification curves for *Gordonclarkvirinae* primers. (**A**) Amplification curves for varying concentrations of phage DNA templates; (**B**) Relative quantification of the templates (normalized to the median concentration group).

**Figure 10:**
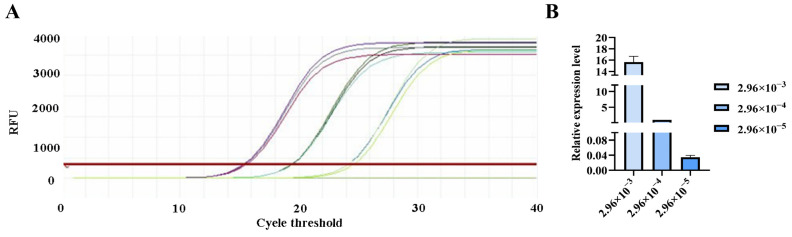
qPCR amplification curves for *Tunavirinae* primers. (**A**) Amplification curves for varying concentrations of phage DNA templates; (**B**) Relative quantification of the templates (normalized to the median concentration group).

**Figure 11:**
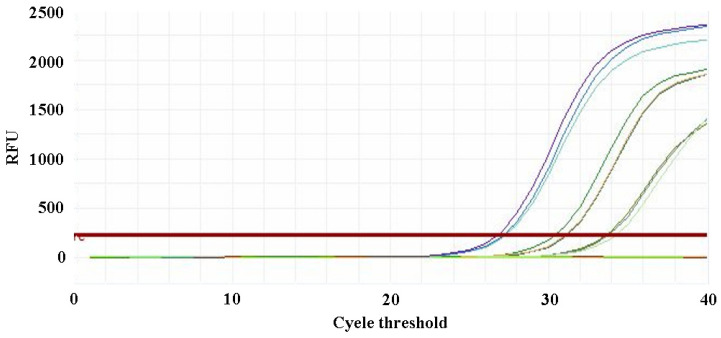
Multiplex qPCR detection of four *Escherichia coli* phages using mixed primer sets. **Note:** Amplification curves for varying concentrations of phage DNA templates.

The qPCR assay was used to evaluate the sensitivity of the detection primers for *Studiervirinae*, *Straboviridae*, *Gordonclarkvirinae*, and *Tunavirinae*. The template DNA concentrations were 4.54 × 10^−3^~10^−5^ μg/μL, 2.08 × 10^−3^~10^−5^ μg/μL, 1.20 × 10^−3^~10^−5^ μg/μL, and 2.96 × 10^−3^~10^−5^ μg/μL, respectively. The results showed that the average Ct values for the *Studiervirinae* primers were 20.24, 24.42, and 27.72; for *Straboviridae*, 19.90, 24.38, and 29.59; for *Gordonclarkvirinae*, 18.20, 22.19, and 26.15; and for *Tunavirinae*, 15.47, 19.44, and 24.30 (Figure 7, Figure 8, Figure 9 and Figure 10). When the four primer pairs were combined for multiplex detection of the four phages using a template DNA dilution factor of 1 × 10^4^, amplification was successfully observed for *Straboviridae*, *Gordonclarkvirinae*, and *Tunavirinae*, with average Ct values of 27.7, 31.18, and 32.44, respectively (Figure 11).

## 4. Discussion

As viruses that specifically infect bacteria, phages possess high host specificity, self-replication capacity, and relative biosafety [18,19]. These unique characteristics endow them with irreplaceable value in diverse applications, including the prevention and control of fermentation contamination, clinical therapy against multidrug-resistant (MDR) bacteria, and biological control in agriculture and aquaculture [1,20]. However, a major bottleneck in phage research and application lies in the lack of efficient detection and screening technologies. The traditional double-layer agar plaque assay relies on visual observation of plaques, which is labor-intensive, time-consuming, and unsuitable for high-throughput screening [21]. Although various approaches developed in recent decades—such as PCR [2,3], Raman spectroscopy [22], ELISA [23], resazurin reduction [24], and mass spectrometry [25]—have been employed for phage detection, each has its limitations. Whole-genome sequencing (WGS) can provide genomic information, but it is complex, time-consuming, and impractical for real-time detection scenarios [26]. Moreover, existing PCR-based methods either target only virulent phages—such as those of Klebsiella pneumoniae and Staphylococcus aureus [27]—or depend on single-gene targets, which fail to detect temperate phages or achieve broad-range detection. These limitations hinder the practical use of phages in contamination surveillance and rapid clinical screening. Therefore, this study proposes a bioinformatics-guided strategy for selecting conserved core genes, combined with PCR and qPCR, to develop a rapid detection system that simultaneously targets both virulent and temperate *Escherichia coli* phages.

In this work, bioinformatics analysis was used to select targets. Among 419 E. coli phage genomes retrieved from the NCBI RefSeq and GenBank databases, vConTACT 2-based classification and ViPTreeGen phylogenetic analysis revealed that these phages could be categorized into 10 families, 20 subfamilies, and 67 genera. BACPHLIP prediction further indicated that virulent phages were concentrated in certain taxa, such as *Straboviridae* and *Demerecviridae*. This finding demonstrated a strong correlation between phage lifestyle (virulent or temperate) and taxonomic grouping. Phages belonging to the same family or subfamily not only clustered phylogenetically but also shared highly conserved functional genes, providing a theoretical basis for selecting core genes. Further pan-genome analysis using Roary and Pirate (with amino acid identity thresholds ranging from 30% to 95%) identified a series of highly conserved core genes at the family, subfamily, and genus levels. Examples include a putative tail tube protein gene in *Ackermannviridae*, the *polA* gene in *Demerecviridae*, and a putative DNA repair helicase gene in *Tunavirinae*. These genes were not randomly selected: (1) they displayed amino acid similarities greater than 60% and average sequence lengths exceeding 400 bp, ensuring suitable conservation and amplification efficiency for primer design; and (2) they were mostly involved in key processes of phage replication (e.g., DNA repair, capsid assembly) or lysis (e.g., lysozyme), exhibiting low horizontal gene transfer (HGT) frequencies, thereby minimizing false negatives due to gene drift. Interestingly, a strong consistency was observed between pan-genomic core gene clustering and phylogenetic relationships, further validating the reliability of the selected detection targets.

From an application standpoint, targeting *E. coli* phages aligns with both public health demands and industrial relevance. Globally, MDR *E. coli* strains account for more than 50% of isolates [1], posing severe challenges to antibiotic therapy, while the host-specific lytic activity of virulent phages offers a promising alternative for treating MDR infections [18,28]. In addition, *E. coli* is widely used in industrial fermentation, where temperate phages cause “latent” contamination. Under stress conditions such as temperature or pH shifts, they can be induced into the lytic cycle, leading to broth clarification, product inactivation, and significant economic losses. The PCR and qPCR systems established in this study effectively address both issues. The PCR assay achieved a detection limit of 10^−5^ μg/μL. Meanwhile, the qPCR system demonstrated high precision, with a coefficient of variation (CV) < 5% across three technical replicates, meeting clinical diagnostic standards. The qPCR assay also enabled real-time quantitative detection, providing valuable data support for phage therapy dose optimization.

In this study, the established phage detection method yielded positive results using four *E. coli* phage strains currently available in the laboratory, obtained from clinical isolates and purchases (vB_EcoP_SP7, EP335, T4, and T7). However, the team is still isolating *E. coli* phages from Xinjiang cattle farms to verify the method’s broad applicability. Additionally, multiplex quantitative PCR successfully amplified targets from *Straboviridae*, *Gordonclarkvirinae*, and *Tunavirinae*, confirming the feasibility of multiplex detection as a rapid screening strategy.

## 5. Conclusions

This study successfully developed a method for rapid molecular typing and detection of *Escherichia coli* phages. By selecting conserved core genes, it achieved an optimal balance between specificity and sensitivity. Based on clade-specific detection, the sensitivity for *Escherichia coli* phages reached 10^−5^ μg/μL, providing a practical tool for the detection of *E. coli* phages.

## Figures and Tables

**Table 1:** Taxonomic distribution of *Escherichia coli* phages (* indicates virulent phages).

Family	Subfamily	Genus	NCBI (incl.ictv)	Average Genome Length, Kbp	Virulent (ncbi.all)
*Escherichia coli* Phages					
*Ackermannviridae*	*Cvivirinae*	*Kuttervirus*	2 (2)	533	2 (2)
		*Taipeivirus*	1 (1)		1 (1)
*Autographiviridae* *	*Molineuxvirinae*	*Vectrevirus*	17 (7)	947	17 (17)
	*Studiervirinae*	*Berlinvirus*	3 (3)	421	3 (3)
		*Kayfunavirus*	22 (5)		22 (22)
		*Przondovirus*	1 (1)		1 (1)
		*Teetrevirus*	3 (1)		3 (3)
		*Teseptimavirus*	4 (2)		4 (4)
		*Ermolevavirus*	1 (1)		1 (1)
*Chaseviridae*	*Cleopatravirinae*	*Carltongylesvirus*	2 (1)	599	2 (2)
*Demerecviridae* *	*Markadamsvirinae*	*Epseptimavirus*	3 (1)	875	3 (3)
		*Tequintavirus*	18 (12)		18 (18)
*Drexlerviridae* *	*Braunvirinae*	*Loudonvirus*	1 (1)		1 (1)
		*Rtpvirus*	2 (2)		2 (2)
	*Rogunavirinae*	*Rogunavirus*	12 (5)		12 (12)
	*Tempevirinae*	*Hanrivervirus*	2 (1)		2 (2)
		*Tlsvirus*	1 (1)		1 (1)
		*Warwickvirus*	2 (0)		2 (2)
	*Tunavirinae*	*Tunavirus*	6 (6)	2015	6 (6)
		*Nouzillyvirus*	1 (1)		1 (1)
*Inoviridae*		*Infulavirus*	2 (1)	1054	2 (2)
*Microviridae*	*Bullavirinae*	*Alphatrevirus*	3 (3)	456	3 (3)
		*Sinsheimervirus*	1 (1)		1 (1)
		*Punavirus*	3 (1)		0 (3)
*Peduoviridae*		*Eganvirus*	1 (1)		0 (1)
		*Peduovirus*	1 (1)		0 (1)
		*Piscesmortuivirus*	1 (1)		1 (1)
*Schitoviridae* *	*Enquatrovirinae*	*Enquatrovirus*	1 (1)		1 (1)
		*Gamaleyavirus*	5 (4)		5 (5)
*Straboviridae* *	*Tevenvirinae*	*Dhakavirus*	8 (5)		8 (8)
		*Gaprivervirus*	5 (4)	924	5 (5)
		*Mosigvirus*	29 (7)		29 (29)
		*Tequatrovirus*	29 (25)		29 (29)
		*Winklervirus*	1 (0)		1 (1)
		*Krischvirus*	10 (4)		10 (10)
	*Gordonclarkvirinae* *	*Kuravirus*	8 (4)	2244	8 (8)
		*Suseptimavirus*	3 (2)		3 (3)
	*Guarnerosvirinae* *	*Mechnikovvirus*	1 (1)	413	1 (1)
		*Jerseyvirus*	1 (0)		1 (1)
		*Kagunavirus*	23 (8)		23 (23)
	*Hendrixvirinae*	*Byrnievirus*	1 (1)	476	0 (1)
		*Cuauhtlivirus*	1 (1)		0 (1)
		*Kwaitsingvirus*	2 (2)		0 (2)
		*Saikungvirus*	1 (1)		0 (1)
		*Shamshuipovirus*	2 (2)		0 (2)
		*Wanchaivirus*	1 (1)		0 (1)
		*Wongtaivirus*	1 (1)		0 (1)
	*Ounavirinae* *	*Felixounavirus*	13 (7)	464	13 (13)
	*Sepvirinae*	*Oslovirus*	5 (2)	2101	0 (5)
		*Traversvirus*	24 (5)		0 (24)
	*Stephanstirmvirinae* *	*Justusliebigvirus*	1 (1)	519	0 (1)
		*Phapecoctavirus*	6 (1)		6 (6)
	*Vequintavirinae* *	*Avunavirus*	1 (1)		1 (1)
		*Seunavirus*	9 (0)		8 (9)
		*Vequintavirus*	6 (3)		6 (6)
		*Dhillonvirus* *	15 (8)	1045	15 (15)
		*Glaedevirus*	1 (0)		0 (1)
		*Lambdavirus*	2 (2)		0 (2)
		*Lederbergvirus*	6 (0)		0 (6)
		*Marienburgvirus*	1 (1)		0 (1)
		*Muvirus*	2 (1)		0 (2)
		*Asteriusvirus* *	7 (2)		7 (7)
		*Ravinvirus*	1 (1)		0 (1)
		*Rosemountvirus*	4 (0)		4 (4)
		*Skarprettervirus*	1 (1)		1 (1)
		*Uetakevirus*	1 (1)		0 (1)
		*Wifcevirus*	2 (2)		2 (2)
Unassigned	Unassigned	Unassigned	62		

**Table 2:** Pan-genome analysis results (* indicates virulent phages).

Family/Subfamily/Genus	Core Gene	Pan-Genome	Similarity
*Ackermannviridae*	*Putative tail tube protein*	Roary	0.95
*Chaseviridae*	*dUTPase*	Roary	0.95
*Demerecviridae* *	*polA*	Roary	0.95
*Inoviridae*	*Hypothetical protein*	Pirate	0.60
*Straboviridae* *	*grcA*	Pirate	0.60
*Molineuxvirinae* *	*DNA ligase*	Roary	0.95
*Studiervirinae* *	*Hypothetical protein*	Pirate	0.60
*Tunavirinae* *	*Putative DNA repair helicase RadD*	Roary	0.95
*Bullavirinae*	*Hypothetical protein*	Pirate	0.70
*Gordonclarkvirinae* *	*Portal protein*	Pirate	0.90
*Guarnerosvirinae* *	*Hypothetical protein*	Roary	0.95
*Hendrixvirinae*	*Hypothetical protein*	Roary	0.95
*Ounavirinae* *	*Lysozyme RrrD*	Roary	0.95
*Sepvirinae*	*Hypothetical protein*	Roary	0.95
*Stephanstirmvirina* *	*Metallophosphatase*	Roary	0.95
*Dhillonvirus* *	*Hypothetical protein*	Roary	0.95

**Table 3:** Primers designed from conserved core genes of *Escherichia coli* phages (* indicates virulent phages).

Family/Subfamily	Primer-F/Primer-R	Product Size (bp)
*Studiervirinaeg* *	F: TTTGACGGAGTGGAGTCCATTGA	73
	R: CTTTGTTTAGGTCCTTCTC	
*Straboviridae* *	F: GAAGATGGTATTCAAGCACGAA	312
	R: TTACAAACTCTCGGTGAAGG	
*Gordonclarkvirinae* *	F: TTGGACGAGTTACGTGTTGACC	435
	R: CGGTGGTACACCTTCAGCTTCT	
*Tunavirinae* *	F: GCTATGGTTGCAAAGCAG	165
	R: CACGATCGGGAAGTAGCA	
*Molineuxvirinae* *	F: ATGCGACCAAACTTCGACTTCGG	854
	R: CCGAACTTAGCCTTGTAAGCACC	
*Chaseviridae*	F: ATGTCTTGCATGACCCAAGTTG	448
	R: CACGACCAGTTGAGCCAAAG	
*Demerecviridae* *	F: ATGAGTAAATCCTGGGGAAAATT	845
	R: GTAAACTTATCTAACACATCTTG	
*Inoviridae*	F: AAAGACGCTCGTTAGCGTTGGT	315
	R: CCCAATTTACGAGCATGAAGAAA	
*Bullavirinae*	F: TGGATGTTACTGAGGAAGAT	259
	R: CGCTCGACGCCATTGATAATGT	
*Guarnerosvirinae* *	F: ATGGGCTACTTTGAGGACTTAAC	354
	R: TTCTACAATCAGCGGCCCCAGG	
*Hendrixvirinae*	F: ATGGTGGAAATCAATAATCAACGT	409
	R: GCTCGAACTGACCATAACCAG	
*Ounavirinae* *	F: ATGCAACTCTCAAGAAAAGG	254
	R: AGTGCATCAAACTCGTTCTGAG	
*Sepvirinae*	F: TGATTGAACTCAGTAATGGACG	665
	R: CAGTCTTCCCACCTGCTGGCAGG	
*Stephanstirmvirinae* *	F: GTTTATTACAAGTGATTTGC	181
	R: TTCTTCAGTCCCGAAACAGAAGT	
*Dhillonvirus* *	F: CATGATTGTTGAACAGCACCGGAC	104
	R: TGGCGCTATAGGTGATGACGTT	

## Data Availability

The original contributions presented in this study are included in the article. Further inquiries can be directed to the corresponding authors. The sequence information in the study are openly available in NCBI with the accession number of PRJNA1393842.

## Supplementary Materials

The following supporting information can be downloaded at: https://www.mdpi.com/article/10.3390/pathogens15010121/s1. Figure S1: Phylogenetic tree of 419 *Escherichia coli* phages (Original figure); Attachment S1: NCBI *Escherichia coli* phage FASTA; Attachment S2: genome_by_genome_overview; Attachment S3: all.bionj.asc.newick; Attachment S4: bacphlip_summary; Attachment S5: Prokka_GFF; Attachment S6: Pan-genome analysis using Roary software; Attachment S7: Pan-genome analysis using Pirate software; Attachment S8: Results of core gene comparison analysis using MAFFT software; Attachment S9: Primer-BLAST verification results of primer specificity.
