# Supplementary material for: Bioinformatics-Driven Systematic Molecular Typing and Rapid qPCR Detection of Escherichia coli Phages: Preliminary Validation with Isolates from Cattle Farms in Xinjiang
Source: Pathogens. 2026 Jan 22;15(1):121. doi: 10.3390/pathogens15010121 (PMC12845203; doi:10.3390/pathogens15010121)
Supplement: Supplementary file 1 [file pathogens-15-00121-s001.zip › Attachment S9 Primer-Blast verification results of primer specificity.zip!/10.Gordonclarkvirinae.pdf]

Edit search New

Input PCR template
none

Specificity of primers
Target templates were found in selected database: Custom (Organism limited to Viruses)

Other reports
Search Summary

Detailed primer reports
You can re-search for specific primers by accepting some of the unintended targets, check the box(es) next to the ones you accept and try again to re-search for specific primers Submit
Help

- Download primer pairs
  - Text
  - CSV
  - Tabular

Primer pair 1

	Sequence (5'->3')	Length	Tm	GC%	Self complementarity	Self 3' complementarity
**Forward primer**	TTGGACGAGTTACGTGTTGACC	22	60.80	50.00	4.00	1.00
**Reverse primer**	CGGTGGTACACCTTCAGCTTCT	22	62.24	54.55	6.00	1.00

Products on intended targets

Products on allowed targets

Products on allowed transcript variants

Products on potentially unintended templates

Products on target templates

>NC_028903.1 Escherichia phage 172-1, complete genome

```
product length = 435
Forward primer  1      TTGGACGAGTTACGTGTTGACC  22
Template        13339  ......................  13360

Reverse primer  1      CGGTGGTACACCTTCAGCTTCT  22
Template        13773  ......................  13752
```

>NC_070984.1 Escherichia phage ES17, complete genome

```
product length = 435
Forward primer  1     TTGGACGAGTTACGTGTTGACC  22
Template        4053  ......................  4074

Reverse primer  1     CGGTGGTACACCTTCAGCTTCT  22
```

```
Template        4487  ......................  4466
```

>NC_070989.1 Escherichia phage vB_EcoP-CHD5UKE1, complete genome

```
product length = 435
Forward primer  1     TTGGACGAGTTACGTGTTGACC  22
Template        3663  ......................  3684

Reverse primer  1     CGGTGGTACACCTTCAGCTTCT  22
Template        4097  ......................  4076
```

>MG673519.1 Escherichia phage LAMP, complete genome

```
product length = 435
Forward primer  1     TTGGACGAGTTACGTGTTGACC  22
Template        3443  ......................  3464

Reverse primer  1     CGGTGGTACACCTTCAGCTTCT  22
Template        3877  ......................  3856
```

>OQ938541.1 Escherichia phage vB_EcoP_PE006, complete genome

```
product length = 435
Forward primer  1     TTGGACGAGTTACGTGTTGACC  22
Template        9962  ......................  9983

Reverse primer  1      CGGTGGTACACCTTCAGCTTCT  22
Template        10396  ......................  10375
```

>PP989838.1 Escherichia phage VTCCBPA_211, complete genome

```
product length = 435
Forward primer  1      TTGGACGAGTTACGTGTTGACC  22
Template        71393  ......................  71372

Reverse primer  1      CGGTGGTACACCTTCAGCTTCT  22
Template        70959  ..................C...  70980
```

>NC_070991.1 Escherichia phage vB_EcoP_WFI101126, complete genome

```
product length = 435
Forward primer  1     TTGGACGAGTTACGTGTTGACC  22
Template        3820  ........A.............  3841

Reverse primer  1     CGGTGGTACACCTTCAGCTTCT  22
Template        4254  ..................C...  4233
```

If you want to allow any of the unintended targets, check the box(es) next to the ones you accept and try again to re-search for specific primers Submit

Help

FOLLOW NCBI
