# Supplementary material for: Bioinformatics-Driven Systematic Molecular Typing and Rapid qPCR Detection of Escherichia coli Phages: Preliminary Validation with Isolates from Cattle Farms in Xinjiang
Source: Pathogens. 2026 Jan 22;15(1):121. doi: 10.3390/pathogens15010121 (PMC12845203; doi:10.3390/pathogens15010121)
Supplement: Supplementary file 1 [file pathogens-15-00121-s001.zip › Attachment S9 Primer-Blast verification results of primer specificity.zip!/11.Guarnerosvirinae.pdf]

Edit search New

Input PCR template
none

Specificity of primers
Target templates were found in selected database: Custom (Organism limited to Viruses)

Other reports
Search Summary

Detailed primer reports + −
You can re-search for specific primers by accepting some of the unintended targets, check the box(es) next to the ones you accept and try again to re-search for specific primers Submit
Help

- Download primer pairs
  - Text
  - CSV
  - Tabular

Primer pair 1

	Sequence (5'->3')	Length	Tm	GC%	Self complementarity	Self 3' complementarity
**Forward primer**	ATGGGCTACTTTGAGGACTTAAC	23	58.16	43.48	4.00	2.00
**Reverse primer**	TTCTACAATCAGCGGCCCCAGG	22	64.62	59.09	4.00	3.00

Products on intended targets

Products on allowed targets

Products on allowed transcript variants

Products on potentially unintended templates

Products on target templates

>KY295896.1 Escherichia phage L_AB-2017, complete genome

```
product length = 354
Forward primer  1     ATGGGCTACTTTGAGGACTTAAC  23
Template        8972  .......................  8950

Reverse primer  1     TTCTACAATCAGCGGCCCCAGG  22
Template        8619  ......................  8640
```

>KY295898.1 Escherichia phage P_AB-2017, complete genome

```
product length = 354
Forward primer  1      ATGGGCTACTTTGAGGACTTAAC  23
Template        16435  .......................  16413

Reverse primer  1      TTCTACAATCAGCGGCCCCAGG  22
```

```
Template         16082  ......................  16103
```

>MZ234048.1 Escherichia phage vB_EcoS-UTI89UKE1, complete genome

```
product length = 354
Forward primer   1      ATGGGCTACTTTGAGGACTTAAC  23
Template         12301  .......................  12323

Reverse primer   1      TTCTACAATCAGCGGCCCCAGG  22
Template         12654  ......................  12633
```

>NC_027993.1 Escherichia phage K1G, complete genome

```
product length = 354
Forward primer   1      ATGGGCTACTTTGAGGACTTAAC  23
Template         10716  .......................  10738

Reverse primer   1      TTCTACAATCAGCGGCCCCAGG  22
Template         11069  ......................  11048
```

>NC_027994.1 Escherichia phage K1H, complete genome

```
product length = 354
Forward primer   1     ATGGGCTACTTTGAGGACTTAAC  23
Template         8831  .......................  8853

Reverse primer   1     TTCTACAATCAGCGGCCCCAGG  22
Template         9184  ......................  9163
```

>NC_041897.1 Escherichia phage K1ind1, complete genome

```
product length = 354
Forward primer   1     ATGGGCTACTTTGAGGACTTAAC  23
Template         9769  .......................  9791

Reverse primer   1      TTCTACAATCAGCGGCCCCAGG  22
Template         10122  ......................  10101
```

>NC_041898.1 Escherichia phage K1ind2, complete genome

```
product length = 354
Forward primer   1     ATGGGCTACTTTGAGGACTTAAC  23
Template         9770  .......................  9792

Reverse primer   1      TTCTACAATCAGCGGCCCCAGG  22
Template         10123  ......................  10102
```

>GU196281.1 Escherichia phage K1ind3, complete genome

```
product length = 354
Forward primer   1      ATGGGCTACTTTGAGGACTTAAC  23
Template         10466  .......................  10488

Reverse primer   1      TTCTACAATCAGCGGCCCCAGG  22
Template         10819  ......................  10798
```

>MG252615.1 Escherichia phage vB_EcoS_HSE2, complete genome

```
product length = 354
Forward primer  1      ATGGGCTACTTTGAGGACTTAAC  23
Template        31453  .......................  31475

Reverse primer  1      TTCTACAATCAGCGGCCCCAGG  22
Template        31806  ......................  31785
```

>MG852086.2 Escherichia phage vB_EcoS-Ro145clw, complete genome

```
product length = 354
Forward primer  1     ATGGGCTACTTTGAGGACTTAAC  23
Template        4173  .......................  4195

Reverse primer  1     TTCTACAATCAGCGGCCCCAGG  22
Template        4526  ......................  4505
```

>MT711525.1 Escherichia phage vB_EcoS_fKuEco01, complete genome

```
product length = 354
Forward primer  1     ATGGGCTACTTTGAGGACTTAAC  23
Template        8615  .......................  8637

Reverse primer  1     TTCTACAATCAGCGGCCCCAGG  22
Template        8968  ......................  8947
```

>MT711527.1 Escherichia phage vB_EcoS_fTaEco01, complete genome

```
product length = 354
Forward primer  1     ATGGGCTACTTTGAGGACTTAAC  23
Template        8614  .......................  8636

Reverse primer  1     TTCTACAATCAGCGGCCCCAGG  22
Template        8967  ......................  8946
```

>MT711528.1 Escherichia phage vB_EcoS_fTaEco03, complete genome

```
product length = 354
Forward primer  1     ATGGGCTACTTTGAGGACTTAAC  23
Template        9053  .......................  9075

Reverse primer  1     TTCTACAATCAGCGGCCCCAGG  22
Template        9406  ......................  9385
```

>MW331437.1 Escherichia phage phiWAO78-1, complete genome

```
product length = 354
Forward primer  1      ATGGGCTACTTTGAGGACTTAAC  23
Template        11647  .......................  11669

Reverse primer  1      TTCTACAATCAGCGGCCCCAGG  22
Template        12000  ......................  11979
```

>NC_073314.1 Escherichia virus fEg-Eco19, complete genome

```
product length = 354
Forward primer  1      ATGGGCTACTTTGAGGACTTAAC  23
Template        16869  .......................  16891

Reverse primer  1      TTCTACAATCAGCGGCCCCAGG  22
Template        17222  ......................  17201
```

>NC_073316.1 Escherichia phage vB_EcoS_fFiEco02, complete genome

```
product length = 354
Forward primer  1      ATGGGCTACTTTGAGGACTTAAC  23
Template        11244  .......................  11266

Reverse primer  1      TTCTACAATCAGCGGCCCCAGG  22
Template        11597  ......................  11576
```

>NC_073324.1 Escherichia phage vB_EcoS-22664BS2, complete genome

```
product length = 354
Forward primer  1      ATGGGCTACTTTGAGGACTTAAC  23
Template        14768  .......................  14790

Reverse primer  1      TTCTACAATCAGCGGCCCCAGG  22
Template        15121  ......................  15100
```

>OM272052.1 Escherichia phage fBC-Eco01, complete genome

```
product length = 354
Forward primer  1      ATGGGCTACTTTGAGGACTTAAC  23
Template        11536  .......................  11558

Reverse primer  1      TTCTACAATCAGCGGCCCCAGG  22
Template        11889  ......................  11868
```

>ON556632.1 Escherichia phage hz69, complete genome

```
product length = 354
Forward primer  1      ATGGGCTACTTTGAGGACTTAAC  23
Template        25503  .......................  25481

Reverse primer  1      TTCTACAATCAGCGGCCCCAGG  22
Template        25150  ......................  25171
```

>OR594180.1 Escherichia phage vB_EcoM_SHAK7693, complete genome

```
product length = 354
Forward primer  1      ATGGGCTACTTTGAGGACTTAAC  23
Template        10728  .......................  10750

Reverse primer  1      TTCTACAATCAGCGGCCCCAGG  22
Template        11081  ......................  11060
```

>OQ442786.1 Escherichia phage JSSK01, complete genome

```
product length = 354
Forward primer  1      ATGGGCTACTTTGAGGACTTAAC  23
```

```
Template        12795  .......................  12817

Reverse primer  1      TTCTACAATCAGCGGCCCCAGG  22
Template        13148  ............T.........  13127
```

>KY295895.1 Escherichia phage G_AB-2017, complete genome

```
product length = 354
Forward primer  1      ATGGGCTACTTTGAGGACTTAAC  23
Template        13534  .....T.................  13512

Reverse primer  1      TTCTACAATCAGCGGCCCCAGG  22
Template        13181  ......................  13202
```

>NC_073317.1 Escherichia phage vB_EcoS_fPoEco01, complete genome

```
product length = 354
Forward primer  1      ATGGGCTACTTTGAGGACTTAAC  23
Template        10869  ..............A........  10891

Reverse primer  1      TTCTACAATCAGCGGCCCCAGG  22
Template        11222  ......................  11201
```

>MK931440.1 Escherichia phage Shashou, complete genome

```
product length = 354
Forward primer  1      ATGGGCTACTTTGAGGACTTAAC  23
Template        10660  ..............A........  10682

Reverse primer  1      TTCTACAATCAGCGGCCCCAGG  22
Template        11013  ......................  10992
```

>NC_073315.1 Escherichia phage vB_EcoS_fFiEco03, complete genome

```
product length = 354
Forward primer  1      ATGGGCTACTTTGAGGACTTAAC  23
Template        10493  ..............A........  10515

Reverse primer  1      TTCTACAATCAGCGGCCCCAGG  22
Template        10846  ......................  10825
```

If you want to allow any of the unintended targets, check the box(es) next to the ones you accept and try again to re-search for specific primers Submit

Help

FOLLOW NCBI

Connect with NLM
