# Supplementary material for: Bioinformatics-Driven Systematic Molecular Typing and Rapid qPCR Detection of Escherichia coli Phages: Preliminary Validation with Isolates from Cattle Farms in Xinjiang
Source: Pathogens. 2026 Jan 22;15(1):121. doi: 10.3390/pathogens15010121 (PMC12845203; doi:10.3390/pathogens15010121)
Supplement: Supplementary file 1 [file pathogens-15-00121-s001.zip › Attachment S9 Primer-Blast verification results of primer specificity.zip!/12.Hendrixvirinae.pdf]

Edit search New

Input PCR template
none

Specificity of primers
Target templates were found in selected database: Custom (Organism limited to Viruses)

Other reports
Search Summary

Detailed primer reports + −
You can re-search for specific primers by accepting some of the unintended targets, check the box(es) next to the ones you accept and try again to re-search for specific primers Submit
Help

- Download primer pairs
  - Text
  - CSV
  - Tabular

Primer pair 1

	Sequence (5'->3')	Length	Tm	GC%	Self complementarity	Self 3' complementarity
**Forward primer**	ATGGTGGAAATCAATAATCAACGT	24	57.23	33.33	4.00	4.00
**Reverse primer**	GCTCGAACTGACCATAACCAG	21	58.73	52.38	4.00	2.00

Products on intended targets

Products on allowed targets

Products on allowed transcript variants

Products on potentially unintended templates

Products on target templates

>NC_019714.1 Enterobacteria phage HK446, complete genome

```
product length = 409
Forward primer  1      ATGGTGGAAATCAATAATCAACGT  24
Template        35802  ........................  35825

Reverse primer  1      GCTCGAACTGACCATAACCAG  21
Template        36210  .....................  36190
```

>NC_019711.1 Enterobacteria phage HK629, complete genome

```
product length = 409
Forward primer  1      ATGGTGGAAATCAATAATCAACGT  24
Template        44090  ........................  44113

Reverse primer  1      GCTCGAACTGACCATAACCAG  21
```

```
Template        44498  .....................  44478
```

>NC_016160.1 Escherichia phage HK75, complete genome

```
product length = 409
Forward primer  1      ATGGTGGAAATCAATAATCAACGT  24
Template        34425  ........................  34448

Reverse primer  1      GCTCGAACTGACCATAACCAG  21
Template        34833  .....................  34813
```

>NC_002166.1 Bacteriophage HK022, complete genome

```
product length = 409
Forward primer  1      ATGGTGGAAATCAATAATCAACGT  24
Template        37574  ........................  37597

Reverse primer  1      GCTCGAACTGACCATAACCAG  21
Template        37982  .....................  37962
```

>NC_019705.1 Enterobacteria phage mEpX2, complete genome

```
product length = 409
Forward primer  1      ATGGTGGAAATCAATAATCAACGT  24
Template        35534  ........................  35557

Reverse primer  1      GCTCGAACTGACCATAACCAG  21
Template        35942  .....................  35922
```

>MG197996.1 Escherichia phage APC_JM3.2, complete genome

```
product length = 409
Forward primer  1     ATGGTGGAAATCAATAATCAACGT  24
Template        8075  ........................  8098

Reverse primer  1     GCTCGAACTGACCATAACCAG  21
Template        8483  .....................  8463
```

>MT833387.1 Escherichia phage HF4s, complete genome

```
product length = 409
Forward primer  1      ATGGTGGAAATCAATAATCAACGT  24
Template        37247  ........................  37270

Reverse primer  1      GCTCGAACTGACCATAACCAG  21
Template        37655  .....................  37635
```

>ON470589.1 Escherichia phage vB_EcoP-611R6, complete genome

```
product length = 409
Forward primer  1      ATGGTGGAAATCAATAATCAACGT  24
Template        20698  ........................  20721

Reverse primer  1      GCTCGAACTGACCATAACCAG  21
Template        21106  .....................  21086
```

>ON470594.1 Escherichia phage vB_EcoP-683R2, complete genome

```
product length = 409
Forward primer  1      ATGGTGGAAATCAATAATCAACGT  24
Template        21775  ........................  21798

Reverse primer  1      GCTCGAACTGACCATAACCAG  21
Template        22183  .....................  22163
```

>NC_002167.1 Enterobacteria phage HK97, complete genome

```
product length = 409
Forward primer  1      ATGGTGGAAATCAATAATCAACGT  24
Template        36552  ........................  36575

Reverse primer  1      GCTCGAACTGACCATAACCAG  21
Template        36960  ..........G..........  36940
```

>NC_019767.1 Enterobacteria phage HK544, complete genome

```
product length = 409
Forward primer  1      ATGGTGGAAATCAATAATCAACGT  24
Template        37232  ........................  37255

Reverse primer  1      GCTCGAACTGACCATAACCAG  21
Template        37640  ..........G..........  37620
```

>MT813197.1 Escherichia phage vB_EcoP_Kapi1, complete genome

```
product length = 409
Forward primer  1      ATGGTGGAAATCAATAATCAACGT  24
Template        16276  ........................  16299

Reverse primer  1      GCTCGAACTGACCATAACCAG  21
Template        16684  ..........G..........  16664
```

>NC_001416.1 Enterobacteria phage lambda, complete genome

```
product length = 409
Forward primer  1      ATGGTGGAAATCAATAATCAACGT  24
Template        45493  .....A..................  45516

Reverse primer  1      GCTCGAACTGACCATAACCAG  21
Template        45901  .....................  45881
```

>NC_019715.1 Enterobacterial phage mEp234, complete genome

```
product length = 409
Forward primer  1      ATGGTGGAAATCAATAATCAACGT  24
Template        36350  .......G................  36373

Reverse primer  1      GCTCGAACTGACCATAACCAG  21
Template        36758  .....................  36738
```

>NC_019769.1 Enterobacteria phage HK542, complete genome

```
product length = 409
Forward primer  1      ATGGTGGAAATCAATAATCAACGT  24
Template        34857  .......G................  34880

Reverse primer  1      GCTCGAACTGACCATAACCAG  21
Template        35265  .....................  35245
```

>MF374379.1 Escherichia phage DN1, complete genome

```
product length = 409
Forward primer  1      ATGGTGGAAATCAATAATCAACGT  24
Template        38550  .....A..................  38573

Reverse primer  1      GCTCGAACTGACCATAACCAG  21
Template        38958  .....................  38938
```

>NC_019709.1 Enterobacteria phage mEpX1, complete genome

```
product length = 409
Forward primer  1      ATGGTGGAAATCAATAATCAACGT  24
Template        37934  ........................  37957

Reverse primer  1      GCTCGAACTGACCATAACCAG  21
Template        38342  ..........G.....G....  38322
```

If you want to allow any of the unintended targets, check the box(es) next to the ones you accept and try again to re-search for specific primers Submit

Help

FOLLOW NCBI

Connect with NLM

National Library of Medicine
8600 Rockville Pike
Bethesda, MD 20894

Web Policies
FOIA
HHS Vulnerability Disclosure

Help
Accessibility
Careers
