# Supplementary material for: Bioinformatics-Driven Systematic Molecular Typing and Rapid qPCR Detection of Escherichia coli Phages: Preliminary Validation with Isolates from Cattle Farms in Xinjiang
Source: Pathogens. 2026 Jan 22;15(1):121. doi: 10.3390/pathogens15010121 (PMC12845203; doi:10.3390/pathogens15010121)
Supplement: Supplementary file 1 [file pathogens-15-00121-s001.zip › Attachment S9 Primer-Blast verification results of primer specificity.zip!/13.Ounavirinae.pdf]

Edit search New

Input PCR template
none

Specificity of primers
Target templates were found in selected database: Custom (Organism limited to Viruses)

Other reports
Search Summary

Detailed primer reports + −
You can re-search for specific primers by accepting some of the unintended targets, check the box(es) next to the ones you accept and try again to re-search for specific primers Submit
Help

- Download primer pairs
  - Text
  - CSV
  - Tabular

Primer pair 1

	Sequence (5'->3')	Length	Tm	GC%	Self complementarity	Self 3' complementarity
**Forward primer**	ATGCAACTCTCAAGAAAAGG	20	53.79	40.00	4.00	0.00
**Reverse primer**	AGTGCATCAAACTCGTTCTGAG	22	58.94	45.45	6.00	4.00

Products on intended targets

Products on allowed targets

Products on allowed transcript variants

Products on potentially unintended templates

Products on target templates

>NC_027337.1 Escherichia phage vB_EcoM-VpaE1, complete genome

```
product length = 254
Forward primer  1      ATGCAACTCTCAAGAAAAGG  20
Template        20938  ....................  20919

Reverse primer  1      AGTGCATCAAACTCGTTCTGAG  22
Template        20685  ......................  20706
```

>NC_028825.1 Escherichia phage vB_EcoM_AYO145A, complete genome

```
product length = 254
Forward primer  1      ATGCAACTCTCAAGAAAAGG  20
Template        21589  ....................  21570

Reverse primer  1      AGTGCATCAAACTCGTTCTGAG  22
```

```
Template        21336  ..............A.......  21357
```

>MH571750.1 Escherichia phage vB_EcoM-Ro111lw, complete genome

```
product length = 254
Forward primer  1     ATGCAACTCTCAAGAAAAGG  20
Template        6324  ....................  6305

Reverse primer  1     AGTGCATCAAACTCGTTCTGAG  22
Template        6071  ........G.............  6092
```

>ON540300.1 Escherichia phage ECOH1, complete genome

```
product length = 254
Forward primer  1     ATGCAACTCTCAAGAAAAGG  20
Template        6314  ....................  6295

Reverse primer  1     AGTGCATCAAACTCGTTCTGAG  22
Template        6061  ........G.............  6082
```

>NC_012749.1 Enterobacteria phage WV8, complete genome

```
product length = 254
Forward primer  1      ATGCAACTCTCAAGAAAAGG  20
Template        21193  ....................  21174

Reverse primer  1      AGTGCATCAAACTCGTTCTGAG  22
Template        20940  .....G........A.......  20961
```

>NC_027369.1 Escherichia phage EC6, complete genome

```
product length = 254
Forward primer  1     ATGCAACTCTCAAGAAAAGG  20
Template        7644  ....................  7625

Reverse primer  1     AGTGCATCAAACTCGTTCTGAG  22
Template        7391  ........G.....A.......  7412
```

>NC_028872.1 Escherichia phage HY02, complete genome

```
product length = 254
Forward primer  1      ATGCAACTCTCAAGAAAAGG  20
Template        20546  ....................  20527

Reverse primer  1      AGTGCATCAAACTCGTTCTGAG  22
Template        20293  ........G.....A.......  20314
```

>NC_029023.1 Escherichia phage JH2, complete genome

```
product length = 254
Forward primer  1      ATGCAACTCTCAAGAAAAGG  20
Template        78991  ....................  79010

Reverse primer  1      AGTGCATCAAACTCGTTCTGAG  22
Template        79244  ........G.....A.......  79223
```

>NC_031082.1 Escherichia phage vB_EcoM_Alf5, complete genome

```
product length = 254
Forward primer  1      ATGCAACTCTCAAGAAAAGG  20
Template        19417  ....................  19398

Reverse primer  1      AGTGCATCAAACTCGTTCTGAG  22
Template        19164  ........G.....A.......  19185
```

>MK482688.1 Escherichia phage vB_EcoM_LMP25, complete genome

```
product length = 254
Forward primer  1      ATGCAACTCTCAAGAAAAGG  20
Template        84047  ....................  84066

Reverse primer  1      AGTGCATCAAACTCGTTCTGAG  22
Template        84300  ........G.....A.......  84279
```

>MK482689.1 Escherichia phage vB_EcoM_LMP33, complete genome

```
product length = 254
Forward primer  1      ATGCAACTCTCAAGAAAAGG  20
Template        69488  ....................  69469

Reverse primer  1      AGTGCATCAAACTCGTTCTGAG  22
Template        69235  ........G.....A.......  69256
```

>MT833282.1 Escherichia coli phage vB_EcoM_Shy, complete genome

```
product length = 254
Forward primer  1      ATGCAACTCTCAAGAAAAGG  20
Template        21996  ....................  21977

Reverse primer  1      AGTGCATCAAACTCGTTCTGAG  22
Template        21743  ........G.....A.......  21764
```

>MZ311863.1 Escherichia phage vB_EcoM_LNA1

```
product length = 254
Forward primer  1      ATGCAACTCTCAAGAAAAGG  20
Template        77708  ....................  77689

Reverse primer  1      AGTGCATCAAACTCGTTCTGAG  22
Template        77455  .....G..G.....A.......  77476
```

If you want to allow any of the unintended targets, check the box(es) next to the ones you accept and try again to re-search for specific primers Submit

Help

FOLLOW NCBI
