# Supplementary material for: Bioinformatics-Driven Systematic Molecular Typing and Rapid qPCR Detection of Escherichia coli Phages: Preliminary Validation with Isolates from Cattle Farms in Xinjiang
Source: Pathogens. 2026 Jan 22;15(1):121. doi: 10.3390/pathogens15010121 (PMC12845203; doi:10.3390/pathogens15010121)
Supplement: Supplementary file 1 [file pathogens-15-00121-s001.zip › Attachment S9 Primer-Blast verification results of primer specificity.zip!/14.Sepvirinae.pdf]

Edit search New

Input PCR template
none

Specificity of primers
Target templates were found in selected database: Custom (Organism limited to Viruses)

Other reports
Search Summary

Detailed primer reports + −

You can re-search for specific primers by accepting some of the unintended targets, check the box(es) next to the ones you accept and try again to re-search for specific primers Submit

Help

- Download primer pairs
  - Text
  - CSV
  - Tabular

Primer pair 1

	Sequence (5'->3')	Length	Tm	GC%	Self complementarity	Self 3' complementarity
**Forward primer**	TGATTGAACTCAGTAATGGACG	22	56.20	40.91	5.00	2.00
**Reverse primer**	CAGTCTTCCCACCTGCTGGCAGG	23	67.29	65.22	8.00	8.00

Products on intended targets

Products on allowed targets

Products on allowed transcript variants

Products on potentially unintended templates

Products on target templates

>NC_028449.1 Escherichia phage PA2, complete genome

```
product length = 665
Forward primer  1      TGATTGAACTCAGTAATGGACG  22
Template        38047  ......................  38068

Reverse primer  1      CAGTCTTCCCACCTGCTGGCAGG  23
Template        38711  .......................  38689
```

>KP682373.1 Escherichia phage PA5, complete genome

```
product length = 665
Forward primer  1      TGATTGAACTCAGTAATGGACG  22
Template        32876  ......................  32897

Reverse primer  1      CAGTCTTCCCACCTGCTGGCAGG  23
```

```
Template        33540  .......................  33518
```

>KP682374.1 Escherichia phage PA8, complete genome

```
product length = 665
Forward primer  1      TGATTGAACTCAGTAATGGACG  22
Template        38062  ......................  38083

Reverse primer  1      CAGTCTTCCCACCTGCTGGCAGG  23
Template        38726  .......................  38704
```

>NC_010237.1 Enterobacteria phage Min27, complete genome

```
product length = 665
Forward primer  1      TGATTGAACTCAGTAATGGACG  22
Template        33749  ......................  33770

Reverse primer  1      CAGTCTTCCCACCTGCTGGCAGG  23
Template        34413  .......................  34391
```

>NC_041935.1 Escherichia phage PA28, complete genome

```
product length = 665
Forward primer  1      TGATTGAACTCAGTAATGGACG  22
Template        35225  ......................  35246

Reverse primer  1      CAGTCTTCCCACCTGCTGGCAGG  23
Template        35889  .......................  35867
```

>NC_049919.1 Escherichia phage SH2026Stx1, complete genome

```
product length = 665
Forward primer  1      TGATTGAACTCAGTAATGGACG  22
Template        34653  ......................  34674

Reverse primer  1      CAGTCTTCCCACCTGCTGGCAGG  23
Template        35317  .......................  35295
```

>MN067333.1 Escherichia phage Lyz12581Vzw, complete genome

```
product length = 665
Forward primer  1     TGATTGAACTCAGTAATGGACG  22
Template        8233  ......................  8212

Reverse primer  1     CAGTCTTCCCACCTGCTGGCAGG  23
Template        7569  .......................  7591
```

>NC_049917.1 Escherichia phage Lys12581Vzw, complete genome

```
product length = 665
Forward primer  1     TGATTGAACTCAGTAATGGACG  22
Template        8233  ......................  8212

Reverse primer  1     CAGTCTTCCCACCTGCTGGCAGG  23
Template        7569  .......................  7591
```

>JQ011318.1 Escherichia phage TL-2011c, complete genome

```
product length = 665
Forward primer  1      TGATTGAACTCAGTAATGGACG  22
Template        34349  ......................  34370

Reverse primer  1      CAGTCTTCCCACCTGCTGGCAGG  23
Template        35013  .......................  34991
```

>NC_028660.1 Escherichia phage phi191, complete genome

```
product length = 665
Forward primer  1      TGATTGAACTCAGTAATGGACG  22
Template        60048  ......................  60027

Reverse primer  1      CAGTCTTCCCACCTGCTGGCAGG  23
Template        59384  .......................  59406
```

>OP797663.1 Escherichia phage 06-3462, complete genome

```
product length = 665
Forward primer  1      TGATTGAACTCAGTAATGGACG  22
Template        35228  ......................  35249

Reverse primer  1      CAGTCTTCCCACCTGCTGGCAGG  23
Template        35892  .......................  35870
```

>KP682372.1 Escherichia phage PA4, complete genome

```
product length = 665
Forward primer  1      TGATTGAACTCAGTAATGGACG  22
Template        32876  ......................  32897

Reverse primer  1      CAGTCTTCCCACCTGCTGGCAGG  23
Template        33540  .......................  33518
```

>KP682375.1 Escherichia phage PA11, complete genome

```
product length = 665
Forward primer  1      TGATTGAACTCAGTAATGGACG  22
Template        32876  ......................  32897

Reverse primer  1      CAGTCTTCCCACCTGCTGGCAGG  23
Template        33540  .......................  33518
```

>KP682376.1 Escherichia phage PA12, complete genome

```
product length = 665
Forward primer  1      TGATTGAACTCAGTAATGGACG  22
Template        31556  ......................  31577

Reverse primer  1      CAGTCTTCCCACCTGCTGGCAGG  23
Template        32220  .......................  32198
```

>KP682377.1 Escherichia phage PA16, complete genome

```
product length = 665
Forward primer  1      TGATTGAACTCAGTAATGGACG  22
Template        32876  ......................  32897

Reverse primer  1      CAGTCTTCCCACCTGCTGGCAGG  23
Template        33540  .......................  33518
```

>KP682378.1 Escherichia phage PA18, complete genome

```
product length = 665
Forward primer  1      TGATTGAACTCAGTAATGGACG  22
Template        32876  ......................  32897

Reverse primer  1      CAGTCTTCCCACCTGCTGGCAGG  23
Template        33540  .......................  33518
```

>KP682379.1 Escherichia phage PA21, complete genome

```
product length = 665
Forward primer  1      TGATTGAACTCAGTAATGGACG  22
Template        32876  ......................  32897

Reverse primer  1      CAGTCTTCCCACCTGCTGGCAGG  23
Template        33540  .......................  33518
```

>KP682380.1 Escherichia phage PA27, complete genome

```
product length = 665
Forward primer  1      TGATTGAACTCAGTAATGGACG  22
Template        34189  ......................  34210

Reverse primer  1      CAGTCTTCCCACCTGCTGGCAGG  23
Template        34853  .......................  34831
```

>KP682382.1 Escherichia phage PA29, complete genome

```
product length = 665
Forward primer  1      TGATTGAACTCAGTAATGGACG  22
Template        32876  ......................  32897

Reverse primer  1      CAGTCTTCCCACCTGCTGGCAGG  23
Template        33540  .......................  33518
```

>KP682383.1 Escherichia phage PA30, complete genome

```
product length = 665
Forward primer  1      TGATTGAACTCAGTAATGGACG  22
Template        32876  ......................  32897

Reverse primer  1      CAGTCTTCCCACCTGCTGGCAGG  23
Template        33540  .......................  33518
```

>KP682384.1 Escherichia phage PA32, complete genome

```
product length = 665
Forward primer  1      TGATTGAACTCAGTAATGGACG  22
```

```
Template        32876  ......................  32897

Reverse primer  1      CAGTCTTCCCACCTGCTGGCAGG  23
Template        33540  .......................  33518
```

>KP682385.1 Escherichia phage PA33, complete genome

```
product length = 665
Forward primer  1      TGATTGAACTCAGTAATGGACG  22
Template        32876  ......................  32897

Reverse primer  1      CAGTCTTCCCACCTGCTGGCAGG  23
Template        33540  .......................  33518
```

>KP682386.1 Escherichia phage PA36, complete genome

```
product length = 665
Forward primer  1      TGATTGAACTCAGTAATGGACG  22
Template        32876  ......................  32897

Reverse primer  1      CAGTCTTCCCACCTGCTGGCAGG  23
Template        33540  .......................  33518
```

>KP682387.1 Escherichia phage PA42, complete genome

```
product length = 665
Forward primer  1      TGATTGAACTCAGTAATGGACG  22
Template        32876  ......................  32897

Reverse primer  1      CAGTCTTCCCACCTGCTGGCAGG  23
Template        33540  .......................  33518
```

>KP682388.1 Escherichia phage PA44, complete genome

```
product length = 665
Forward primer  1      TGATTGAACTCAGTAATGGACG  22
Template        32876  ......................  32897

Reverse primer  1      CAGTCTTCCCACCTGCTGGCAGG  23
Template        33540  .......................  33518
```

>KP682389.1 Escherichia phage PA45, complete genome

```
product length = 665
Forward primer  1      TGATTGAACTCAGTAATGGACG  22
Template        35502  ......................  35523

Reverse primer  1      CAGTCTTCCCACCTGCTGGCAGG  23
Template        36166  .......................  36144
```

>KP682390.1 Escherichia phage PA50, complete genome

```
product length = 665
Forward primer  1      TGATTGAACTCAGTAATGGACG  22
Template        32876  ......................  32897

Reverse primer  1      CAGTCTTCCCACCTGCTGGCAGG  23
```

```
Template        33540  .......................  33518
```

>KP682391.1 Escherichia phage PA51, complete genome

```
product length = 665
Forward primer  1      TGATTGAACTCAGTAATGGACG  22
Template        31556  ......................  31577

Reverse primer  1      CAGTCTTCCCACCTGCTGGCAGG  23
Template        32220  .......................  32198
```

>KP682392.1 Escherichia phage PA52, complete genome

```
product length = 665
Forward primer  1      TGATTGAACTCAGTAATGGACG  22
Template        34189  ......................  34210

Reverse primer  1      CAGTCTTCCCACCTGCTGGCAGG  23
Template        34853  .......................  34831
```

>NC_000924.1 Enterobacteria phage 933W, complete genome

```
product length = 665
Forward primer  1      TGATTGAACTCAGTAATGGACG  22
Template        32070  ......................  32091

Reverse primer  1      CAGTCTTCCCACCTGCTGGCAGG  23
Template        32734  .......................  32712
```

If you want to allow any of the unintended targets, check the box(es) next to the ones you accept and try again to re-search for specific primers Submit

Help

FOLLOW NCBI

Connect with NLM

National Library of Medicine
8600 Rockville Pike
Bethesda, MD 20894

Web Policies
FOIA
HHS Vulnerability Disclosure
