# Supplementary material for: Bioinformatics-Driven Systematic Molecular Typing and Rapid qPCR Detection of Escherichia coli Phages: Preliminary Validation with Isolates from Cattle Farms in Xinjiang
Source: Pathogens. 2026 Jan 22;15(1):121. doi: 10.3390/pathogens15010121 (PMC12845203; doi:10.3390/pathogens15010121)
Supplement: Supplementary file 1 [file pathogens-15-00121-s001.zip › Attachment S9 Primer-Blast verification results of primer specificity.zip!/15.Stephanstirmvirinae.pdf]

Edit search New

Input PCR template
none

Specificity of primers
Target templates were found in selected database: Custom (Organism limited to Viruses)

Other reports
Search Summary

Detailed primer reports + −
You can re-search for specific primers by accepting some of the unintended targets, check the box(es) next to the ones you accept and try again to re-search for specific primers Submit
Help

- Download primer pairs
  - Text
  - CSV
  - Tabular

Primer pair 1

	Sequence (5'->3')	Length	Tm	GC%	Self complementarity	Self 3' complementarity
**Forward primer**	GTTTATTACAAGTGATTTGC	20	48.64	30.00	5.00	3.00
**Reverse primer**	TTCTTCAGTCCCGAAACAGAAGT	23	59.87	43.48	8.00	6.00

Products on intended targets

Products on allowed targets

Products on allowed transcript variants

Products on potentially unintended templates

Products on target templates

>NC_023693.1 Enterobacteria phage phi92, complete genome

```
product length = 181
Forward primer  1      GTTTATTACAAGTGATTTGC  20
Template        45282  ....................  45263

Reverse primer  1      TTCTTCAGTCCCGAAACAGAAGT  23
Template        45102  .......................  45124
```

>NC_020079.1 Escherichia phage phAPEC8, complete genome

```
product length = 181
Forward primer  1      GTTTATTACAAGTGATTTGC  20
Template        54536  ....................  54517

Reverse primer  1      TTCTTCAGTCCCGAAACAGAAGT  23
```

```
Template        54356  .......................  54378


product length = 3502
Forward primer  1      GTTTATTACAAGTGATTTGC  20
Template        57857  T.......A..A.T....T.  57838

Reverse primer  1      TTCTTCAGTCCCGAAACAGAAGT  23
Template        54356  .......................  54378
```

>MZ726793.1 Escherichia phage vB_EcoM_ASO2A, complete genome

```
product length = 181
Forward primer  1      GTTTATTACAAGTGATTTGC  20
Template        56530  ....................  56511

Reverse primer  1      TTCTTCAGTCCCGAAACAGAAGT  23
Template        56350  .......................  56372


product length = 3520
Forward primer  1      GTTTATTACAAGTGATTTGC  20
Template        59869  T.......A..A.T....T.  59850

Reverse primer  1      TTCTTCAGTCCCGAAACAGAAGT  23
Template        56350  .......................  56372
```

>OM362897.1 Escherichia phage SKA64, complete genome

```
product length = 181
Forward primer  1      GTTTATTACAAGTGATTTGC  20
Template        10735  ............C.......  10754

Reverse primer  1      TTCTTCAGTCCCGAAACAGAAGT  23
Template        10915  .......................  10893
```

>OL505078.1 Escherichia phage BI-EHEC, complete genome

```
product length = 181
Forward primer  1      GTTTATTACAAGTGATTTGC  20
Template        51616  ............C.......  51597

Reverse primer  1      TTCTTCAGTCCCGAAACAGAAGT  23
Template        51436  .......................  51458


product length = 3523
Forward primer  1      GTTTATTACAAGTGATTTGC  20
Template        54958  T.......A..A.T....T.  54939

Reverse primer  1      TTCTTCAGTCCCGAAACAGAAGT  23
Template        51436  .......................  51458
```

>OL739525.1 Escherichia phage dw-ec, complete genome

```
product length = 181
Forward primer  1      GTTTATTACAAGTGATTTGC  20
Template        50425  ............C.......  50406
```

```
Reverse primer  1       TTCTTCAGTCCCGAAACAGAAGT  23
Template        50245   .......................  50267
```

>OR786310.1 Escherichia phage ETEC-TG, complete genome

```
product length = 181
Forward primer  1       GTTTATTACAAGTGATTTGC  20
Template        62563   ............C.......  62544

Reverse primer  1       TTCTTCAGTCCCGAAACAGAAGT  23
Template        62383   .......................  62405


product length = 3522
Forward primer  1       GTTTATTACAAGTGATTTGC  20
Template        65904   T.......A..A.T....T.  65885

Reverse primer  1       TTCTTCAGTCCCGAAACAGAAGT  23
Template        62383   .......................  62405
```

>NC_054919.1 Escherichia phage vB_EcoM_G4507, complete genome

```
product length = 1002
Forward primer  1       GTTTATTACAAGTGATTTGC  20
Template        69729   TC.C..........G....  69748

Forward primer  1       GTTTATTACAAGTGATTTGC  20
Template        70730   .C..T...A.TC........  70711
```

If you want to allow any of the unintended targets, check the box(es) next to the ones you accept and try again to re-search for specific primers Submit

Help

FOLLOW NCBI

Connect with NLM

National Library of Medicine
8600 Rockville Pike
Bethesda, MD 20894

Web Policies
FOIA
HHS Vulnerability Disclosure
