# Supplementary material for: Bioinformatics-Driven Systematic Molecular Typing and Rapid qPCR Detection of Escherichia coli Phages: Preliminary Validation with Isolates from Cattle Farms in Xinjiang
Source: Pathogens. 2026 Jan 22;15(1):121. doi: 10.3390/pathogens15010121 (PMC12845203; doi:10.3390/pathogens15010121)
Supplement: Supplementary file 1 [file pathogens-15-00121-s001.zip › Attachment S9 Primer-Blast verification results of primer specificity.zip!/16.Dhillonvirus.pdf]

Edit search New

Input PCR template
none

Specificity of primers
Target templates were found in selected database: Custom (Organism limited to Viruses)

Other reports
Search Summary

Detailed primer reports +−
You can re-search for specific primers by accepting some of the unintended targets, check the box(es) next to the ones you accept and try again to re-search for specific primers Submit
Help

- Download primer pairs
  - Text
  - CSV
  - Tabular

Primer pair 1

	Sequence (5'->3')	Length	Tm	GC%	Self complementarity	Self 3' complementarity
**Forward primer**	CATGATTGTTGAACAGCACCGGAC	24	62.90	50.00	7.00	1.00
**Reverse primer**	TGGCGCTATAGGTGATGACGTT	22	61.85	50.00	6.00	2.00

Products on intended targets

Products on allowed targets

Products on allowed transcript variants

Products on potentially unintended templates

Products on target templates

>NC_073060.1 Escherichia phage vB_EcoS_WF5505, complete genome

```
product length = 104
Forward primer  1     CATGATTGTTGAACAGCACCGGAC  24
Template        4875  ........................  4898

Reverse primer  1     TGGCGCTATAGGTGATGACGTT  22
Template        4978  ......................  4957
```

>NC_073061.1 Escherichia phage vB_EcoS-101114BS4, complete genome

```
product length = 104
Forward primer  1     CATGATTGTTGAACAGCACCGGAC  24
Template        3149  ........................  3172

Reverse primer  1     TGGCGCTATAGGTGATGACGTT  22
```

```
Template        3252  ......................  3231
```

>MK373791.1 Escherichia phage vB_EcoS_WFI, complete genome

```
product length = 104
Forward primer  1     CATGATTGTTGAACAGCACCGGAC  24
Template        4960  ........................  4983

Reverse primer  1     TGGCGCTATAGGTGATGACGTT  22
Template        5063  ......................  5042
```

>MZ234008.1 Escherichia phage vB_EcoS-22664B1, complete genome

```
product length = 104
Forward primer  1     CATGATTGTTGAACAGCACCGGAC  24
Template        4515  ........................  4538

Reverse primer  1     TGGCGCTATAGGTGATGACGTT  22
Template        4618  ......................  4597
```

>MZ234014.1 Escherichia phage vB_EcoS-101114B2, complete genome

```
product length = 104
Forward primer  1     CATGATTGTTGAACAGCACCGGAC  24
Template        4430  ........................  4453

Reverse primer  1     TGGCGCTATAGGTGATGACGTT  22
Template        4533  ......................  4512
```

>MZ234026.1 Escherichia phage vB_EcoS-CHD2B1, complete genome

```
product length = 104
Forward primer  1      CATGATTGTTGAACAGCACCGGAC  24
Template        40708  ........................  40685

Reverse primer  1      TGGCGCTATAGGTGATGACGTT  22
Template        40605  ......................  40626
```

>NC_073058.1 Escherichia phage vB_EcoS_WFI, complete genome

```
product length = 104
Forward primer  1     CATGATTGTTGAACAGCACCGGAC  24
Template        4960  ........................  4983

Reverse primer  1     TGGCGCTATAGGTGATGACGTT  22
Template        5063  ......................  5042
```

>PP786691.1 Escherichia phage phiEco273, complete genome

```
product length = 104
Forward primer  1      CATGATTGTTGAACAGCACCGGAC  24
Template        44578  ........................  44555

Reverse primer  1      TGGCGCTATAGGTGATGACGTT  22
Template        44475  ......................  44496
```

>NC_073064.1 Escherichia phage vB_EcoS_PTXU06, complete genome

```
product length = 104
Forward primer  1     CATGATTGTTGAACAGCACCGGAC  24
Template        4516  ........................  4539

Reverse primer  1     TGGCGCTATAGGTGATGACGTT  22
Template        4619  ................T.....  4598
```

>NC_073059.1 Escherichia phage vB_EcoS-CHD5UKE2, complete genome

```
product length = 104
Forward primer  1      CATGATTGTTGAACAGCACCGGAC  24
Template        13101  .........C..............  13078

Reverse primer  1      TGGCGCTATAGGTGATGACGTT  22
Template        12998  ......................  13019
```

>NC_027383.1 Escherichia phage YD-2008.s, complete genome

```
product length = 104
Forward primer  1      CATGATTGTTGAACAGCACCGGAC  24
Template        31667  ........................  31644

Reverse primer  1      TGGCGCTATAGGTGATGACGTT  22
Template        31564  .......G.....A........  31585
```

>NC_073078.1 Escherichia phage welsh, complete genome

```
product length = 104
Forward primer  1      CATGATTGTTGAACAGCACCGGAC  24
Template        33957  ........................  33980

Reverse primer  1      TGGCGCTATAGGTGATGACGTT  22
Template        34060  .......G.....A........  34039
```

>JQ086375.1 Enterobacteria phage HK578, complete genome

```
product length = 104
Forward primer  1      CATGATTGTTGAACAGCACCGGAC  24
Template        43283  ...................A....  43306

Reverse primer  1      TGGCGCTATAGGTGATGACGTT  22
Template        43386  .......G.....A........  43365
```

>NC_024783.1 Enterobacteria phage EK99P-1, complete genome

```
product length = 104
Forward primer  1      CATGATTGTTGAACAGCACCGGAC  24
Template        44125  ...................A....  44148

Reverse primer  1      TGGCGCTATAGGTGATGACGTT  22
Template        44228  .......G..T...........  44207
```

>PP627283.1 Escherichia phage SM_S22, complete genome

```
product length = 104
Forward primer  1      CATGATTGTTGAACAGCACCGGAC  24
Template        44537  ......A.................  44560

Reverse primer  1      TGGCGCTATAGGTGATGACGTT  22
Template        44640  .......G.....A........  44619
```

>NC_041898.1 Escherichia phage K1ind2, complete genome

```
product length = 104
Forward primer  1      CATGATTGTTGAACAGCACCGGAC  24
Template        12570  ...............A.....C..  12547

Reverse primer  1      TGGCGCTATAGGTGATGACGTT  22
Template        12467  C.........A..A..T.....  12488
```

>GU196281.1 Escherichia phage K1ind3, complete genome

```
product length = 104
Forward primer  1      CATGATTGTTGAACAGCACCGGAC  24
Template        13266  ...............A.....C..  13243

Reverse primer  1      TGGCGCTATAGGTGATGACGTT  22
Template        13163  C.........A..A..T.....  13184
```

>MK931440.1 Escherichia phage Shashou, complete genome

```
product length = 104
Forward primer  1      CATGATTGTTGAACAGCACCGGAC  24
Template        13007  ...............A.....C..  12984

Reverse primer  1      TGGCGCTATAGGTGATGACGTT  22
Template        12904  C.........A..A..T..A..  12925
```

>KY295895.1 Escherichia phage G_AB-2017, complete genome

```
product length = 104
Forward primer  1      CATGATTGTTGAACAGCACCGGAC  24
Template        10734  ............G..A.....C..  10757

Reverse primer  1      TGGCGCTATAGGTGATGACGTT  22
Template        10837  C.........A..A..T.....  10816
```

>MZ234048.1 Escherichia phage vB_EcoS-UTI89UKE1, complete genome

```
product length = 104
Forward primer  1      CATGATTGTTGAACAGCACCGGAC  24
Template        15101  ............G..A.....C..  15078

Reverse primer  1      TGGCGCTATAGGTGATGACGTT  22
Template        14998  C.........A..A..T.....  15019
```

>NC_027994.1 Escherichia phage K1H, complete genome

```
product length = 104
Forward primer  1      CATGATTGTTGAACAGCACCGGAC  24
```

```
Template        11631  ............G..A.....C..  11608

Reverse primer  1      TGGCGCTATAGGTGATGACGTT  22
Template        11528  C.........A..A..T.....  11549
```

>NC_041897.1 Escherichia phage K1ind1, complete genome

```
product length = 104
Forward primer  1      CATGATTGTTGAACAGCACCGGAC  24
Template        12569  ............G..A.....C..  12546

Reverse primer  1      TGGCGCTATAGGTGATGACGTT  22
Template        12466  C.........A..A..T.....  12487
```

>MT711525.1 Escherichia phage vB_EcoS_fKuEco01, complete genome

```
product length = 104
Forward primer  1      CATGATTGTTGAACAGCACCGGAC  24
Template        11415  ............G..A.....C..  11392

Reverse primer  1      TGGCGCTATAGGTGATGACGTT  22
Template        11312  C.........A..A..T.....  11333
```

>MT711527.1 Escherichia phage vB_EcoS_fTaEco01, complete genome

```
product length = 104
Forward primer  1      CATGATTGTTGAACAGCACCGGAC  24
Template        11414  ............G..A.....C..  11391

Reverse primer  1      TGGCGCTATAGGTGATGACGTT  22
Template        11311  C.........A..A..T.....  11332
```

>MT711528.1 Escherichia phage vB_EcoS_fTaEco03, complete genome

```
product length = 104
Forward primer  1      CATGATTGTTGAACAGCACCGGAC  24
Template        11853  ............G..A.....C..  11830

Reverse primer  1      TGGCGCTATAGGTGATGACGTT  22
Template        11750  C.........A..A..T.....  11771
```

>MW331437.1 Escherichia phage phiWAO78-1, complete genome

```
product length = 104
Forward primer  1      CATGATTGTTGAACAGCACCGGAC  24
Template        14448  ............G..A.....C..  14425

Reverse primer  1      TGGCGCTATAGGTGATGACGTT  22
Template        14345  ..........A..A..T..A..  14366
```

>NC_073316.1 Escherichia phage vB_EcoS_fFiEco02, complete genome

```
product length = 104
Forward primer  1      CATGATTGTTGAACAGCACCGGAC  24
Template        14165  ............G..A.....C..  14142

Reverse primer  1      TGGCGCTATAGGTGATGACGTT  22
```

```
Template        14062  C.........A..A..T.....  14083
```

>KY295896.1 Escherichia phage L_AB-2017, complete genome

```
product length = 104
Forward primer  1     CATGATTGTTGAACAGCACCGGAC  24
Template        6169  ............G..A.....C..  6192

Reverse primer  1     TGGCGCTATAGGTGATGACGTT  22
Template        6272  C.........A..A..T..A..  6251
```

>NC_073317.1 Escherichia phage vB_EcoS_fPoEco01, complete genome

```
product length = 104
Forward primer  1      CATGATTGTTGAACAGCACCGGAC  24
Template        13790  ............G..A.....C..  13767

Reverse primer  1      TGGCGCTATAGGTGATGACGTT  22
Template        13687  C.........A..A..T..A..  13708
```

>NC_073314.1 Escherichia virus fEg-Eco19, complete genome

```
product length = 104
Forward primer  1      CATGATTGTTGAACAGCACCGGAC  24
Template        19790  ............G..A.....C..  19767

Reverse primer  1      TGGCGCTATAGGTGATGACGTT  22
Template        19687  C...A.......A..T..A..  19708
```

>NC_073315.1 Escherichia phage vB_EcoS_fFiEco03, complete genome

```
product length = 104
Forward primer  1      CATGATTGTTGAACAGCACCGGAC  24
Template        13414  ............G..A.....C..  13391

Reverse primer  1      TGGCGCTATAGGTGATGACGTT  22
Template        13311  C.........A..A..T..A..  13332
```

>NC_073324.1 Escherichia phage vB_EcoS-22664BS2, complete genome

```
product length = 104
Forward primer  1      CATGATTGTTGAACAGCACCGGAC  24
Template        17571  ............G..A.....C..  17548

Reverse primer  1      TGGCGCTATAGGTGATGACGTT  22
Template        17468  C.........A..A..T..A..  17489
```

>OM272052.1 Escherichia phage fBC-Eco01, complete genome

```
product length = 104
Forward primer  1      CATGATTGTTGAACAGCACCGGAC  24
Template        13883  ............G..A.....C..  13860

Reverse primer  1      TGGCGCTATAGGTGATGACGTT  22
Template        13780  C...A.......A..T..A..  13801
```

>ON556632.1 Escherichia phage hz69, complete genome

```
product length = 104
Forward primer  1      CATGATTGTTGAACAGCACCGGAC  24
Template        23185  ............G..A.....C..  23208

Reverse primer  1      TGGCGCTATAGGTGATGACGTT  22
Template        23288  C.........A..A..T..A..  23267
```

>OQ442786.1 Escherichia phage JSSK01, complete genome

```
product length = 104
Forward primer  1      CATGATTGTTGAACAGCACCGGAC  24
Template        15577  ............G..A.....C..  15554

Reverse primer  1      TGGCGCTATAGGTGATGACGTT  22
Template        15474  C.........A..A..T..A..  15495
```

>OR594180.1 Escherichia phage vB_EcoM_SHAK7693, complete genome

```
product length = 104
Forward primer  1      CATGATTGTTGAACAGCACCGGAC  24
Template        13077  ............G..A.....C..  13054

Reverse primer  1      TGGCGCTATAGGTGATGACGTT  22
Template        12974  C...A.........A..T..A..  12995
```

>KY295898.1 Escherichia phage P_AB-2017, complete genome

```
product length = 104
Forward primer  1      CATGATTGTTGAACAGCACCGGAC  24
Template        13635  ....G.......G..A..T..C..  13658

Reverse primer  1      TGGCGCTATAGGTGATGACGTT  22
Template        13738  C.........A..A..T.....  13717
```

>NC_027993.1 Escherichia phage K1G, complete genome

```
product length = 104
Forward primer  1      CATGATTGTTGAACAGCACCGGAC  24
Template        13516  ....G.......G..A..T..C..  13493

Reverse primer  1      TGGCGCTATAGGTGATGACGTT  22
Template        13413  C.........A..A..T.....  13434
```

If you want to allow any of the unintended targets, check the box(es) next to the ones you accept and try again to re-search for specific primers Submit

Help

FOLLOW NCBI
