# Supplementary material for: Bioinformatics-Driven Systematic Molecular Typing and Rapid qPCR Detection of Escherichia coli Phages: Preliminary Validation with Isolates from Cattle Farms in Xinjiang
Source: Pathogens. 2026 Jan 22;15(1):121. doi: 10.3390/pathogens15010121 (PMC12845203; doi:10.3390/pathogens15010121)
Supplement: Supplementary file 1 [file pathogens-15-00121-s001.zip › Attachment S9 Primer-Blast verification results of primer specificity.zip!/2.Molineuxvirinae.pdf]

Edit search New

Input PCR template
none

Specificity of primers
Target templates were found in selected database: Custom

Other reports
Search Summary

Detailed primer reports
You can re-search for specific primers by accepting some of the unintended targets, check the box(es) next to the ones you accept and try again to re-search for specific primers Submit
Help

- Download primer pairs
  - Text
  - CSV
  - Tabular

Primer pair 1

	Sequence (5'->3')	Length	Tm	GC%	Self complementarity	Self 3' complementarity
**Forward primer**	ATGCGACCAAACTTCGACTTCGG	23	63.77	52.17	4.00	4.00
**Reverse primer**	CCGAACTTAGCCTTGTAAGCACC	23	61.72	52.17	4.00	0.00

Products on intended targets

Products on allowed targets

Products on allowed transcript variants

Products on potentially unintended templates

Products on target templates

>NC_008152.1 Escherichia phage K1-5, complete genome

```
product length = 854
Forward primer  1      ATGCGACCAAACTTCGACTTCGG  23
Template        15337  .......................  15359

Reverse primer  1      CCGAACTTAGCCTTGTAAGCACC  23
Template        16190  .......................  16168
```

>NC_047769.1 Escherichia phage AAPEc6, complete genome

```
product length = 854
Forward primer  1      ATGCGACCAAACTTCGACTTCGG  23
Template        15034  .......................  15056

Reverse primer  1      CCGAACTTAGCCTTGTAAGCACC  23
```

```
Template        15887  .......................  15865
```

>NC_047806.1 Escherichia phage vB_EcoP_B, complete genome

```
product length = 854
Forward primer  1     ATGCGACCAAACTTCGACTTCGG  23
Template        6637  .......................  6615

Reverse primer  1     CCGAACTTAGCCTTGTAAGCACC  23
Template        5784  .......................  5806
```

>NC_047807.1 Escherichia phage vB_EcoP_C, complete genome

```
product length = 854
Forward primer  1      ATGCGACCAAACTTCGACTTCGG  23
Template        31779  .......................  31757

Reverse primer  1      CCGAACTTAGCCTTGTAAGCACC  23
Template        30926  .......................  30948
```

>NC_047809.1 Escherichia phage vB_EcoP_K, complete genome

```
product length = 854
Forward primer  1      ATGCGACCAAACTTCGACTTCGG  23
Template        28730  .......................  28752

Reverse primer  1      CCGAACTTAGCCTTGTAAGCACC  23
Template        29583  .......................  29561
```

>NC_047899.1 Escherichia phage VEc3, complete genome

```
product length = 854
Forward primer  1      ATGCGACCAAACTTCGACTTCGG  23
Template        15654  .......................  15676

Reverse primer  1      CCGAACTTAGCCTTGTAAGCACC  23
Template        16507  .......................  16485
```

>KY295893.1 Escherichia phage vB_EcoP_D, complete genome

```
product length = 854
Forward primer  1     ATGCGACCAAACTTCGACTTCGG  23
Template        7978  .......................  7956

Reverse primer  1     CCGAACTTAGCCTTGTAAGCACC  23
Template        7125  .......................  7147
```

>KY295899.1 Escherichia phage vB_EcoP_R, complete genome

```
product length = 854
Forward primer  1     ATGCGACCAAACTTCGACTTCGG  23
Template        9693  .......................  9671

Reverse primer  1     CCGAACTTAGCCTTGTAAGCACC  23
Template        8840  .......................  8862
```

>MK373771.1 Escherichia phage vB_EcoP_R4596, complete genome

```
product length = 854
Forward primer  1      ATGCGACCAAACTTCGACTTCGG  23
Template        15132  .......................  15154

Reverse primer  1      CCGAACTTAGCCTTGTAAGCACC  23
Template        15985  .......................  15963
```

>MZ234012.1 Escherichia phage vB_EcoP-101101UKE1, complete genome

```
product length = 854
Forward primer  1      ATGCGACCAAACTTCGACTTCGG  23
Template        13293  .......................  13315

Reverse primer  1      CCGAACTTAGCCTTGTAAGCACC  23
Template        14146  .......................  14124
```

>MZ234019.1 Escherichia phage vB_EcoP-101117UKE2, complete genome

```
product length = 854
Forward primer  1      ATGCGACCAAACTTCGACTTCGG  23
Template        12780  .......................  12802

Reverse primer  1      CCGAACTTAGCCTTGTAAGCACC  23
Template        13633  .......................  13611
```

>MZ234020.1 Escherichia phage vB_EcoP-101118B1, complete genome

```
product length = 854
Forward primer  1      ATGCGACCAAACTTCGACTTCGG  23
Template        12725  .......................  12747

Reverse primer  1      CCGAACTTAGCCTTGTAAGCACC  23
Template        13578  .......................  13556
```

>MZ234032.1 Escherichia phage vB_EcoP-G3A1, complete genome

```
product length = 854
Forward primer  1      ATGCGACCAAACTTCGACTTCGG  23
Template        12725  .......................  12747

Reverse primer  1      CCGAACTTAGCCTTGTAAGCACC  23
Template        13578  .......................  13556
```

>MZ234049.1 Escherichia phage vB_EcoP-UTI89UKE2, complete genome

```
product length = 854
Forward primer  1      ATGCGACCAAACTTCGACTTCGG  23
Template        13134  .......................  13156

Reverse primer  1      CCGAACTTAGCCTTGTAAGCACC  23
Template        13987  ...........T...........  13965
```

>MZ234050.1 Escherichia phage vB_EcoP-UTI89UKE3, complete genome

```
product length = 854
Forward primer  1      ATGCGACCAAACTTCGACTTCGG  23
Template        13135  .......................  13157

Reverse primer  1      CCGAACTTAGCCTTGTAAGCACC  23
Template        13988  ...........T...........  13966
```

>NC_047889.1 Escherichia phage mutPK1A2, complete genome

```
product length = 854
Forward primer  1      ATGCGACCAAACTTCGACTTCGG  23
Template        15772  .......................  15794

Reverse primer  1      CCGAACTTAGCCTTGTAAGCACC  23
Template        16625  ...........T........T..  16603
```

>MK373773.1 Escherichia phage vB_EcoP_KAW1A4500, complete genome

```
product length = 854
Forward primer  1      ATGCGACCAAACTTCGACTTCGG  23
Template        15981  .......................  16003

Reverse primer  1      CCGAACTTAGCCTTGTAAGCACC  23
Template        16834  ..............A..T.....  16812
```

If you want to allow any of the unintended targets, check the box(es) next to the ones you accept and try again to re-search for specific primers Submit

Help

FOLLOW NCBI

Connect with NLM

National Library of Medicine
8600 Rockville Pike
Bethesda, MD 20894

Web Policies
FOIA
HHS Vulnerability Disclosure

Help
Accessibility
Careers
