# Supplementary material for: Bioinformatics-Driven Systematic Molecular Typing and Rapid qPCR Detection of Escherichia coli Phages: Preliminary Validation with Isolates from Cattle Farms in Xinjiang
Source: Pathogens. 2026 Jan 22;15(1):121. doi: 10.3390/pathogens15010121 (PMC12845203; doi:10.3390/pathogens15010121)
Supplement: Supplementary file 1 [file pathogens-15-00121-s001.zip › Attachment S9 Primer-Blast verification results of primer specificity.zip!/3.Studiervirinaeg(73bp).pdf]

Edit search New

Input PCR template
none

Specificity of primers
Target templates were found in selected database: Custom (Organism limited to Viruses)

Other reports
Search Summary

Detailed primer reports + −
You can re-search for specific primers by accepting some of the unintended targets, check the box(es) next to the ones you accept and try again to re-search for specific primers Submit
Help

- Download primer pairs
  - Text
  - CSV
  - Tabular

Primer pair 1

	Sequence (5'->3')	Length	Tm	GC%	Self complementarity	Self 3' complementarity
**Forward primer**	AACGTCCTGAAGTATCGACT	20	56.02	45.00	4.00	2.00
**Reverse primer**	CTTTGTTTAGGTCCTTCTC	19	50.32	42.11	5.00	1.00

Products on intended targets

Products on allowed targets

Products on allowed transcript variants

Products on potentially unintended templates

Products on target templates

>NC_047927.1 Escherichia phage YZ1, complete genome

```
product length = 73
Forward primer  1     AACGTCCTGAAGTATCGACT  20
Template        7567  ....................  7586

Reverse primer  1     CTTTGTTTAGGTCCTTCTC  19
Template        7639  ...................  7621
```

>MZ311862.1 Escherichia phage vB_EcoP_LNA3

```
product length = 73
Forward primer  1      AACGTCCTGAAGTATCGACT  20
Template        24468  ....................  24487

Reverse primer  1      CTTTGTTTAGGTCCTTCTC  19
```

```
Template        24540  ...................  24522
```

>OQ703102.1 Escherichia phage vB_EcoP-R1, complete genome

```
product length = 73
Forward primer  1      AACGTCCTGAAGTATCGACT  20
Template        26528  ....................  26547

Reverse primer  1      CTTTGTTTAGGTCCTTCTC  19
Template        26600  ...................  26582
```

>NC_047808.1 Escherichia phage vB_EcoP_F, complete genome

```
product length = 73
Forward primer  1      AACGTCCTGAAGTATCGACT  20
Template        15070  ....................  15089

Reverse primer  1      CTTTGTTTAGGTCCTTCTC  19
Template        15142  .......C...........  15124
```

>KX689784.2 Escherichia phage JSS1, complete genome

```
product length = 73
Forward primer  1      AACGTCCTGAAGTATCGACT  20
Template        31202  ....................  31183

Reverse primer  1      CTTTGTTTAGGTCCTTCTC  19
Template        31130  .......C...........  31148
```

>MZ234011.1 Escherichia phage vB_EcoP-22664UKE3-2, complete genome

```
product length = 73
Forward primer  1      AACGTCCTGAAGTATCGACT  20
Template        18910  ....................  18929

Reverse primer  1      CTTTGTTTAGGTCCTTCTC  19
Template        18982  .......C...........  18964
```

>MZ234022.1 Escherichia phage vB_EcoP-101120B1-2, complete genome

```
product length = 73
Forward primer  1      AACGTCCTGAAGTATCGACT  20
Template        18692  ....................  18711

Reverse primer  1      CTTTGTTTAGGTCCTTCTC  19
Template        18764  .......C...........  18746
```

>MZ234023.1 Escherichia phage vB_EcoP-101120B2, complete genome

```
product length = 73
Forward primer  1      AACGTCCTGAAGTATCGACT  20
Template        18747  ....................  18766

Reverse primer  1      CTTTGTTTAGGTCCTTCTC  19
Template        18819  .......C...........  18801
```

>MZ234024.1 Escherichia phage vB_EcoP-101136BS1, complete genome

```
product length = 73
Forward primer  1      AACGTCCTGAAGTATCGACT  20
Template        17677  ....................  17696

Reverse primer  1      CTTTGTTTAGGTCCTTCTC  19
Template        17749  .......C...........  17731
```

>MZ234044.1 Escherichia phage vB_EcoP-PM129, complete genome

```
product length = 73
Forward primer  1      AACGTCCTGAAGTATCGACT  20
Template        19041  ....................  19060

Reverse primer  1      CTTTGTTTAGGTCCTTCTC  19
Template        19113  .......C...........  19095
```

>MZ234045.1 Escherichia phage vB_EcoP-PM131, complete genome

```
product length = 73
Forward primer  1      AACGTCCTGAAGTATCGACT  20
Template        19041  ....................  19060

Reverse primer  1      CTTTGTTTAGGTCCTTCTC  19
Template        19113  .......C...........  19095
```

>MZ234046.1 Escherichia phage vB_EcoP-PM137, complete genome

```
product length = 73
Forward primer  1      AACGTCCTGAAGTATCGACT  20
Template        19041  ....................  19060

Reverse primer  1      CTTTGTTTAGGTCCTTCTC  19
Template        19113  .......C...........  19095
```

>MZ234047.1 Escherichia phage vB_EcoP-PM139, complete genome

```
product length = 73
Forward primer  1      AACGTCCTGAAGTATCGACT  20
Template        19041  ....................  19060

Reverse primer  1      CTTTGTTTAGGTCCTTCTC  19
Template        19113  .......C...........  19095
```

>MZ311861.1 Escherichia phage vB_EcoP_LNA5

```
product length = 73
Forward primer  1     AACGTCCTGAAGTATCGACT  20
Template        9136  ....................  9117

Reverse primer  1     CTTTGTTTAGGTCCTTCTC  19
Template        9064  .......C...........  9082
```

>NC_047777.1 Escherichia phage ZG49, complete genome

```
product length = 73
Forward primer  1     AACGTCCTGAAGTATCGACT  20
Template        8302  ....................  8321

Reverse primer  1     CTTTGTTTAGGTCCTTCTC  19
Template        8374  .......C...........  8356
```

```
product length = 524
Forward primer  1     AACGTCCTGAAGTATCGACT  20
Template        8302  ....................  8321

Reverse primer  1     CTTTGTTTAGGTCCTTCTC  19
Template        8825  .......C...........  8807
```

```
product length = 73
Forward primer  1     AACGTCCTGAAGTATCGACT  20
Template        8753  ....................  8772

Reverse primer  1     CTTTGTTTAGGTCCTTCTC  19
Template        8825  .......C...........  8807
```

>KC109329.1 Escherichia phage CLB_P1, complete genome

```
product length = 73
Forward primer  1     AACGTCCTGAAGTATCGACT  20
Template        8096  ........T...........  8115

Reverse primer  1     CTTTGTTTAGGTCCTTCTC  19
Template        8168  ...................  8150
```

>MK903279.1 Escherichia phage Peacock, complete genome

```
product length = 73
Forward primer  1     AACGTCCTGAAGTATCGACT  20
Template        8649  ....................  8668

Reverse primer  1     CTTTGTTTAGGTCCTTCTC  19
Template        8721  .......C.A.........  8703
```

>MZ234009.1 Escherichia phage vB_EcoP-22664BS1, complete genome

```
product length = 73
Forward primer  1      AACGTCCTGAAGTATCGACT  20
Template        17658  ........T...........  17677

Reverse primer  1      CTTTGTTTAGGTCCTTCTC  19
Template        17730  .......C...........  17712
```

>MZ234021.1 Escherichia phage vB_EcoP-101118UKE1, complete genome

```
product length = 73
Forward primer  1      AACGTCCTGAAGTATCGACT  20
Template        18076  ...............A....  18095

Reverse primer  1      CTTTGTTTAGGTCCTTCTC  19
Template        18148  .......C...........  18130
```

>NC_048023.1 Escherichia phage Vec13, complete genome

```
product length = 73
Forward primer  1     AACGTCCTGAAGTATCGACT  20
Template        8264  ....................  8283

Reverse primer  1     CTTTGTTTAGGTCCTTCTC  19
Template        8336  ....CGCC.T.........  8318
```

>NC_015249.1 Enterobacteria phage 285P, complete genome

```
product length = 73
Forward primer  1     AACGTCCTGAAGTATCGACT  20
Template        8544  ...A....T........C..  8563

Reverse primer  1     CTTTGTTTAGGTCCTTCTC  19
Template        8616  ....CGCC.T.........  8598
```

>NC_028822.1 Escherichia phage P483, complete genome

```
product length = 73
Forward primer  1      AACGTCCTGAAGTATCGACT  20
Template        27998  ...A....T........C..  27979

Reverse primer  1      CTTTGTTTAGGTCCTTCTC  19
Template        27926  ....CGCC.T.........  27944
```

>MT740314.1 Escherichia phage JEP1, complete genome

```
product length = 3044
Reverse primer  1     CTTTGTTTAGGTCCTTCTC  19
Template        7422  .C.C...G.A.C.......  7404

Reverse primer  1     CTTTGTTTAGGTCCTTCTC  19
Template        4379  G..GA.......G..A...  4397
```

>NC_005859.1 Enterobacteria phage T5, complete genome

```
product length = 3708
Forward primer  1      AACGTCCTGAAGTATCGACT  20
Template        97918  ..TTCT..........T...  97899

Reverse primer  1      CTTTGTTTAGGTCCTTCTC  19
Template        94211  ...GTC....AA.......  94229
```

>NC_020079.1 Escherichia phage phAPEC8, complete genome

```
product length = 3873
Reverse primer  1      CTTTGTTTAGGTCCTTCTC  19
Template        10273  A........AAG.T.....  10255

Reverse primer  1     CTTTGTTTAGGTCCTTCTC  19
Template        6401  A.A....G..CA.......  6419
```

>AY587007.1 Bacteriophage T5 strain ATCC 11303-B5, complete genome

```
product length = 3708
Forward primer  1      AACGTCCTGAAGTATCGACT  20
Template        98000  ..TTCT..........T...  97981

Reverse primer  1      CTTTGTTTAGGTCCTTCTC  19
Template        94293  ...GTC....AA.......  94311
```

>AY692264.1 Bacteriophage T5 strain st0 deletion mutant, complete genome

```
product length = 3708
Forward primer  1      AACGTCCTGAAGTATCGACT  20
Template        89876  ..TTCT..........T...  89857

Reverse primer  1      CTTTGTTTAGGTCCTTCTC  19
Template        86169  ...GTC....AA.......  86187
```

>PP554575.1 Escherichia phage PMBT16, complete genome

```
product length = 3040
Reverse primer  1       CTTTGTTTAGGTCCTTCTC  19
Template        112913  .C.C...G.A.C.......  112895

Reverse primer  1       CTTTGTTTAGGTCCTTCTC  19
Template        109874  G..GA.......G..A...  109892
```

>MK373771.1 Escherichia phage vB_EcoP_R4596, complete genome

```
product length = 2800
Forward primer  1      AACGTCCTGAAGTATCGACT  20
Template        17935  ..T.CTT..........T..  17954

Reverse primer  1      CTTTGTTTAGGTCCTTCTC  19
Template        20734  A.G.C.....CG.......  20716
```

If you want to allow any of the unintended targets, check the box(es) next to the ones you accept and try again to re-search for specific primers Submit

Help

FOLLOW NCBI

Connect with NLM

National Library of Medicine
8600 Rockville Pike
Bethesda, MD 20894
