# Supplementary material for: Bioinformatics-Driven Systematic Molecular Typing and Rapid qPCR Detection of Escherichia coli Phages: Preliminary Validation with Isolates from Cattle Farms in Xinjiang
Source: Pathogens. 2026 Jan 22;15(1):121. doi: 10.3390/pathogens15010121 (PMC12845203; doi:10.3390/pathogens15010121)
Supplement: Supplementary file 1 [file pathogens-15-00121-s001.zip › Attachment S9 Primer-Blast verification results of primer specificity.zip!/5.Demerecviridae.pdf]

Edit search New

Input PCR template
none

Specificity of primers
Target templates were found in selected database: Custom (Organism limited to Viruses)

Other reports
Search Summary

Detailed primer reports

You can re-search for specific primers by accepting some of the unintended targets, check the box(es) next to the ones you accept and try again to re-search for specific primers Submit

Help

- Download primer pairs
  - Text
  - CSV
  - Tabular

Primer pair 1

	Sequence (5'->3')	Length	Tm	GC%	Self complementarity	Self 3' complementarity
Forward primer	ATGAGTAAATCCTGGGGAAAATT	23	55.84	34.78	4.00	4.00
Reverse primer	GTAAACTTATCTAACACATCTTG	23	51.12	30.43	4.00	1.00

Products on intended targets

Products on allowed targets

Products on allowed transcript variants

Products on potentially unintended templates

Products on target templates

>NC_005859.1 Enterobacteria phage T5, complete genome

```
product length = 845
Forward primer  1      ATGAGTAAATCCTGGGGAAAATT  23
Template        80093  .......................  80115

Reverse primer  1      GTAAACTTATCTAACACATCTTG  23
Template        80937  .......................  80915
```

>AY587007.1 Bacteriophage T5 strain ATCC 11303-B5, complete genome

```
product length = 845
Forward primer  1      ATGAGTAAATCCTGGGGAAAATT  23
Template        80177  .......................  80199

Reverse primer  1      GTAAACTTATCTAACACATCTTG  23
```

```
Template        81021  .......................  80999
```

>AY692264.1 Bacteriophage T5 strain st0 deletion mutant, complete genome

```
product length = 845
Forward primer  1      ATGAGTAAATCCTGGGGAAAATT  23
Template        72053  .......................  72075

Reverse primer  1      GTAAACTTATCTAACACATCTTG  23
Template        72897  .......................  72875
```

>MK373797.1 Escherichia phage vB_EcoS_HASG4, complete genome

```
product length = 845
Forward primer  1      ATGAGTAAATCCTGGGGAAAATT  23
Template        80203  .......................  80225

Reverse primer  1      GTAAACTTATCTAACACATCTTG  23
Template        81047  ..............T........  81025
```

>NC_017969.1 Escherichia phage vB_EcoS_AKFV33, complete genome

```
product length = 845
Forward primer  1      ATGAGTAAATCCTGGGGAAAATT  23
Template        78204  .......................  78226

Reverse primer  1      GTAAACTTATCTAACACATCTTG  23
Template        79048  ..............T........  79026
```

>NC_048748.1 Escherichia phage vB_EcoS_HdH2, complete genome

```
product length = 845
Forward primer  1      ATGAGTAAATCCTGGGGAAAATT  23
Template        79105  .......................  79127

Reverse primer  1      GTAAACTTATCTAACACATCTTG  23
Template        79949  ..............T........  79927
```

>MK373799.1 Escherichia phage vB_EcoS_EASG3, complete genome

```
product length = 845
Forward primer  1      ATGAGTAAATCCTGGGGAAAATT  23
Template        80160  .......................  80182

Reverse primer  1      GTAAACTTATCTAACACATCTTG  23
Template        81004  ..............T........  80982
```

>OR515477.1 Escherichia phage Es2, complete genome

```
product length = 845
Forward primer  1      ATGAGTAAATCCTGGGGAAAATT  23
Template        95576  .......................  95554

Reverse primer  1      GTAAACTTATCTAACACATCTTG  23
Template        94732  ..............T........  94754
```

>MT843274.1 Escherichia phage BB1, complete genome

```
product length = 845
Forward primer  1      ATGAGTAAATCCTGGGGAAAATT  23
Template        48091  .......................  48113

Reverse primer  1      GTAAACTTATCTAACACATCTTG  23
Template        48935  ..............T..G.....  48913
```

>NC_027356.1 Escherichia phage DT57C, complete genome

```
product length = 845
Forward primer  1      ATGAGTAAATCCTGGGGAAAATT  23
Template        68007  .......................  68029

Reverse primer  1      GTAAACTTATCTAACACATCTTG  23
Template        68851  ..............T..G.....  68829
```

>NC_047749.1 Enterobacteria phage DT571/2, complete genome

```
product length = 845
Forward primer  1      ATGAGTAAATCCTGGGGAAAATT  23
Template        68536  .......................  68558

Reverse primer  1      GTAAACTTATCTAACACATCTTG  23
Template        69380  ..............T..G.....  69358
```

>NC_047979.1 Escherichia phage Gostya9, complete genome

```
product length = 845
Forward primer  1      ATGAGTAAATCCTGGGGAAAATT  23
Template        72441  .......................  72463

Reverse primer  1      GTAAACTTATCTAACACATCTTG  23
Template        73285  ..............T..G.....  73263
```

>NC_048731.1 Escherichia phage fp01, complete genome

```
product length = 845
Forward primer  1      ATGAGTAAATCCTGGGGAAAATT  23
Template        55401  .......................  55423

Reverse primer  1      GTAAACTTATCTAACACATCTTG  23
Template        56245  .....T........T........  56223
```

>MT833283.1 Escherichia coli phage vB_EcoS_Ace, complete genome

```
product length = 845
Forward primer  1      ATGAGTAAATCCTGGGGAAAATT  23
Template        82178  .......................  82200

Reverse primer  1      GTAAACTTATCTAACACATCTTG  23
Template        83022  ..............T..G.....  83000
```

>MF431737.1 Escherichia phage saus132, complete genome

```
product length = 845
Forward primer  1      ATGAGTAAATCCTGGGGAAAATT  23
Template        84708  .......................  84730

Reverse primer  1      GTAAACTTATCTAACACATCTTG  23
Template        85552  .............GT..G.....  85530
```

>NC_042307.1 , *** SEQUENCING IN PROGRESS ***

```
product length = 845
Forward primer  1      ATGAGTAAATCCTGGGGAAAATT  23
Template        68389  .......................  68411

Reverse primer  1      GTAAACTTATCTAACACATCTTG  23
Template        69233  ..........T...T..G.....  69211
```

>NC_047884.1 Bacteriophage T5-like saus132, complete genome

```
product length = 845
Forward primer  1      ATGAGTAAATCCTGGGGAAAATT  23
Template        84708  .......................  84730

Reverse primer  1      GTAAACTTATCTAACACATCTTG  23
Template        85552  .............GT..G.....  85530
```

>MF431730.1 Escherichia phage chee24, complete genome

```
product length = 845
Forward primer  1      ATGAGTAAATCCTGGGGAAAATT  23
Template        79579  .....C.................  79601

Reverse primer  1      GTAAACTTATCTAACACATCTTG  23
Template        80423  .....T........T........  80401
```

>NC_048818.1 Escherichia phage VEc33, complete genome

```
product length = 845
Forward primer  1      ATGAGTAAATCCTGGGGAAAATT  23
Template        79846  .....C.................  79868

Reverse primer  1      GTAAACTTATCTAACACATCTTG  23
Template        80690  ..............T..G.....  80668
```

>NC_047822.1 Escherichia phage phiLLS, complete genome

```
product length = 845
Forward primer  1     ATGAGTAAATCCTGGGGAAAATT  23
Template        3437  .....C.................  3415

Reverse primer  1     GTAAACTTATCTAACACATCTTG  23
Template        2593  ..............T..G.....  2615
```

>NC_047885.1 Bacteriophage T5-like chee24, complete genome

```
product length = 845
Forward primer  1      ATGAGTAAATCCTGGGGAAAATT  23
```

```
Template        79579  .....C.................  79601

Reverse primer  1      GTAAACTTATCTAACACATCTTG  23
Template        80423  .....T........T........  80401
```

If you want to allow any of the unintended targets, check the box(es) next to the ones you accept and try again to re-search for specific primers Submit

Help

FOLLOW NCBI

Connect with NLM

National Library of Medicine
8600 Rockville Pike
Bethesda, MD 20894

Web Policies
FOIA
HHS Vulnerability Disclosure

Help
Accessibility
Careers

NLM NIH HHS USA.gov
