# Supplementary material for: Bioinformatics-Driven Systematic Molecular Typing and Rapid qPCR Detection of Escherichia coli Phages: Preliminary Validation with Isolates from Cattle Farms in Xinjiang
Source: Pathogens. 2026 Jan 22;15(1):121. doi: 10.3390/pathogens15010121 (PMC12845203; doi:10.3390/pathogens15010121)
Supplement: Supplementary file 1 [file pathogens-15-00121-s001.zip › Attachment S9 Primer-Blast verification results of primer specificity.zip!/6.Tunavirinae.pdf]

Target templates were found in selected database: Nucleotide collection (nt)

Other reports

Search Summary

Detailed primer reports

You can re-search for specific primers by accepting some of the unintended targets, check the box(es) next to the ones you accept and try again to re-search for specific primers Submit

Help

- Download primer pairs
  - Text
  - CSV
  - Tabular

Primer pair 1

	Sequence (5'->3')	Length	Tm	GC%	Self complementarity	Self 3' complementarity
**Forward primer**	GCTATGGTTGCAAAGCAG	18	54.48	50.00	6.00	2.00
**Reverse primer**	CACGATCGGGAAGTAGCA	18	56.46	55.56	6.00	0.00

Products on intended targets

Products on allowed targets

Products on allowed transcript variants

Products on potentially unintended templates

Products on target templates

>NC_049832.1 Escherichia phage vB_EcoS-DELF2 DNA, complete genome

```
product length = 165
Forward primer  1       GCTATGGTTGCAAAGCAG  18
Template        29678   ..................  29661

Reverse primer  1       CACGATCGGGAAGTAGCA  18
Template        29514   ..................  29531
```

```
product length = 3736
Forward primer  1      GCTATGGTTGCAAAGCAG  18
Template        14569  .......G.........C  14552

Reverse primer  1      CACGATCGGGAAGTAGCA  18
Template        10834  AT.....A..........  10851
```

>OL960582.1 Escherichia phage vB_EcoS_SA12KD, complete genome

```
product length = 165
Forward primer  1      GCTATGGTTGCAAAGCAG  18
Template        27952  ..................  27969

Reverse primer  1      CACGATCGGGAAGTAGCA  18
Template        28116  ..................  28099
```

>OL960579.1 Escherichia phage vB_EcoS_SA30RD, complete genome

```
product length = 165
Forward primer  1      GCTATGGTTGCAAAGCAG  18
Template        28109  ..................  28126

Reverse primer  1      CACGATCGGGAAGTAGCA  18
Template        28273  ..................  28256
```

>OL770108.1 Escherichia phage CLB_P3, complete genome

```
product length = 165
Forward primer  1     GCTATGGTTGCAAAGCAG  18
Template        7270  ........G.........  7253

Reverse primer  1     CACGATCGGGAAGTAGCA  18
Template        7106  ..................  7123
```

>OL960578.1 Escherichia phage vB_EcoS_SA32RD, complete genome

```
product length = 165
Forward primer  1      GCTATGGTTGCAAAGCAG  18
```

```
Template        27616  ........G.........  27633

Reverse primer  1      CACGATCGGGAAGTAGCA  18
Template        27780  ..................  27763
```

>NC_005833.1 Escherichia phage T1, complete genome

```
product length = 165
Forward primer  1      GCTATGGTTGCAAAGCAG  18
Template        36726  ........G.........  36743

Reverse primer  1      CACGATCGGGAAGTAGCA  18
Template        36890  ..................  36873


product length = 2941
Forward primer  1     GCTATGGTTGCAAAGCAG  18
Template        1843  ..A....GA........C  1860

Reverse primer  1     CACGATCGGGAAGTAGCA  18
Template        4783  AT.....A..........  4766
```

>OM362897.1 Escherichia phage SKA64, complete genome

```
product length = 3051
Forward primer  1       GCTATGGTTGCAAAGCAG  18
Template        130068  .A.....GGC.......C  130051

Reverse primer  1       CACGATCGGGAAGTAGCA  18
Template        127018  ...C...T.T........  127035
```

>OP557969.1 Escherichia phage ph0011, complete genome

```
product length = 1336
Forward primer  1       GCTATGGTTGCAAAGCAG  18
Template        336550  AAA..............A  336567

Reverse primer  1       CACGATCGGGAAGTAGCA  18
Template        337885  .CAC...A........A.  337868
```

>MK327934.1 Escherichia phage vB_EcoM_G2469, complete genome

```
product length = 1075
Forward primer  1       GCTATGGTTGCAAAGCAG  18
Template        135949  .G.C...GA........T  135966

Forward primer  1       GCTATGGTTGCAAAGCAG  18
Template        137023  ..CG.....CA.......  137006
```

>NC_023693.1 Enterobacteria phage phi92, complete genome

```
product length = 441
Forward primer  1      GCTATGGTTGCAAAGCAG  18
Template        24205  ...C..T.GC.......C  24222

Forward primer  1      GCTATGGTTGCAAAGCAG  18
Template        24645  C....AA..C........  24628
```

>NC_070984.1 Escherichia phage ES17, complete genome

```
product length = 1559
Reverse primer  1      CACGATCGGGAAGTAGCA  18
Template        53514  GTA.....T........T  53497

Reverse primer  1      CACGATCGGGAAGTAGCA  18
Template        51956  G.T.C..........G.  51973
```

>MZ234015.1 Escherichia phage vB_EcoP-101114BS3, complete genome

```
product length = 2309
Forward primer  1     GCTATGGTTGCAAAGCAG  18
Template        8314  AT.T..C.A.........  8331

Reverse primer  1      CACGATCGGGAAGTAGCA  18
Template        10622  AT.T...CT.........  10605


product length = 3377
Forward primer  1     GCTATGGTTGCAAAGCAG  18
```

```
Template        8314  AT.T..C.A.........  8331

Forward primer  1      GCTATGGTTGCAAAGCAG  18
Template        11690  TT.G..T..........A  11673
```

>NC_070982.1 Escherichia phage vB_EcoP-101114UKE3, complete genome

```
product length = 2309
Forward primer  1      GCTATGGTTGCAAAGCAG  18
Template        29861  AT.T..C.A.........  29878

Reverse primer  1      CACGATCGGGAAGTAGCA  18
Template        32169  AT.T...CT.........  32152


product length = 3377
Forward primer  1      GCTATGGTTGCAAAGCAG  18
Template        29861  AT.T..C.A.........  29878

Forward primer  1      GCTATGGTTGCAAAGCAG  18
Template        33237  TT.G..T..........A  33220
```

>NC_018859.1 Escherichia phage ECBP2, complete genome

```
product length = 2863
Forward primer  1      GCTATGGTTGCAAAGCAG  18
Template        30226  A..T..C.AA........  30243

Reverse primer  1      CACGATCGGGAAGTAGCA  18
Template        33088  AT.T...CT.........  33071


product length = 3934
Forward primer  1      GCTATGGTTGCAAAGCAG  18
Template        30226  A..T..C.AA........  30243

Forward primer  1      GCTATGGTTGCAAAGCAG  18
Template        34159  TT.G..T..........A  34142
```

>MN158207.1 Escherichia phage BEK3, complete genome

```
product length = 2988
Forward primer  1      GCTATGGTTGCAAAGCAG  18
Template        27784  TGA.......A...T...  27801

Forward primer  1      GCTATGGTTGCAAAGCAG  18
Template        30771  ..G.ATA.C.........  30754
```

>MN158206.1 Escherichia phage BEK2, complete genome

```
product length = 2987
Forward primer  1      GCTATGGTTGCAAAGCAG  18
Template        27781  TGA.......A...T...  27798

Forward primer  1      GCTATGGTTGCAAAGCAG  18
Template        30767  ..G.ATA.C.........  30750
```

>OQ689734.1 Escherichia phage 4E8, complete genome

```
product length = 2819
Forward primer  1      GCTATGGTTGCAAAGCAG  18
Template        30243  AT.T..C.A.........  30260

Reverse primer  1      CACGATCGGGAAGTAGCA  18
Template        33061  AT.T...CT.........  33044


product length = 3044
Forward primer  1      GCTATGGTTGCAAAGCAG  18
Template        30243  AT.T..C.A.........  30260

Forward primer  1      GCTATGGTTGCAAAGCAG  18
Template        33286  A.GG...A.T........  33269


product length = 2212
Forward primer  1      GCTATGGTTGCAAAGCAG  18
Template        30243  AT.T..C.A.........  30260

Forward primer  1      GCTATGGTTGCAAAGCAG  18
Template        32454  .TAT....A.......T.  32437
```

If you want to allow any of the unintended targets, check the box(es) next to the ones you accept and try again to re-search for specific primers

Submit

Help

FOLLOW NCBI

Connect with NLM

National Library of Medicine
8600 Rockville Pike
Bethesda, MD 20894

Web Policies
FOIA
HHS Vulnerability Disclosure

Help
Accessibility
Careers

NLM NIH HHS USA.gov
