# Supplementary material for: Bioinformatics-Driven Systematic Molecular Typing and Rapid qPCR Detection of Escherichia coli Phages: Preliminary Validation with Isolates from Cattle Farms in Xinjiang
Source: Pathogens. 2026 Jan 22;15(1):121. doi: 10.3390/pathogens15010121 (PMC12845203; doi:10.3390/pathogens15010121)
Supplement: Supplementary file 1 [file pathogens-15-00121-s001.zip › Attachment S9 Primer-Blast verification results of primer specificity.zip!/8.Bullavirinae.pdf]

Edit search New

Input PCR template
none

Specificity of primers
Target templates were found in selected database: Custom (Organism limited to Viruses)

Other reports
Search Summary

Detailed primer reports + −
You can re-search for specific primers by accepting some of the unintended targets, check the box(es) next to the ones you accept and try again to re-search for specific primers Submit
Help

- Download primer pairs
  - Text
  - CSV
  - Tabular

Primer pair 1

	Sequence (5'->3')	Length	Tm	GC%	Self complementarity	Self 3' complementarity
**Forward primer**	TGGATGTTACTGAGGAAGAT	20	53.02	40.00	7.00	7.00
**Reverse primer**	CGCTCGACGCCATTGATAATGT	22	61.68	50.00	6.00	4.00

Products on intended targets

Products on allowed targets

Products on allowed transcript variants

Products on potentially unintended templates

Products on target templates

>NC_001330.1 Enterobacteria phage alpha3, complete genome

```
product length = 259
Forward primer  1     TGGATGTTACTGAGGAAGAT  20
Template        2576  ....................  2595

Reverse primer  1     CGCTCGACGCCATTGATAATGT  22
Template        2834  ......................  2813
```

>NC_012868.1 Enterobacteria phage St-1, complete genome

```
product length = 259
Forward primer  1     TGGATGTTACTGAGGAAGAT  20
Template        2575  .....A.C............  2594

Reverse primer  1     CGCTCGACGCCATTGATAATGT  22
```

```
Template        2833  ..............A.......  2812
```

>NC_001730.1 Bacteriophage phiK complete genome

```
product length = 259
Forward primer  1     TGGATGTTACTGAGGAAGAT  20
Template        2575  .....A.C..C.........  2594

Reverse primer  1     CGCTCGACGCCATTGATAATGT  22
Template        2833  ..............A.......  2812
```

>NC_012741.1 Enterobacteria phage JS10, complete genome

```
product length = 919
Forward primer  1       TGGATGTTACTGAGGAAGAT  20
Template        107606  .......GGT...T.....A  107625

Forward primer  1       TGGATGTTACTGAGGAAGAT  20
Template        108524  .CCG.......A.A......  108505
```

>OL770107.1 Escherichia phage CLB_P2, complete genome

```
product length = 915
Forward primer  1       TGGATGTTACTGAGGAAGAT  20
Template        151537  .CCG.......A.A......  151556

Forward primer  1       TGGATGTTACTGAGGAAGAT  20
Template        152451  .......GGT...T.....A  152432
```

If you want to allow any of the unintended targets, check the box(es) next to the ones you accept and try again to re-search for specific primers Submit

Help

FOLLOW NCBI

Connect with NLM

National Library of Medicine
8600 Rockville Pike
Bethesda, MD 20894

Web Policies
FOIA
HHS Vulnerability Disclosure
