# Supplementary material for: Bioinformatics-Driven Systematic Molecular Typing and Rapid qPCR Detection of Escherichia coli Phages: Preliminary Validation with Isolates from Cattle Farms in Xinjiang
Source: Pathogens. 2026 Jan 22;15(1):121. doi: 10.3390/pathogens15010121 (PMC12845203; doi:10.3390/pathogens15010121)
Supplement: Supplementary file 1 [file pathogens-15-00121-s001.zip › Attachment S9 Primer-Blast verification results of primer specificity.zip!/9.Straboviridae(A).pdf]

Edit search New

Input PCR template
none

Specificity of primers
Target templates were found in selected database: Custom (Organism limited to Viruses)

Other reports
Search Summary

Detailed primer reports + −
You can re-search for specific primers by accepting some of the unintended targets, check the box(es) next to the ones you accept and try again to re-search for specific primers Submit
Help

- Download primer pairs
  - Text
  - CSV
  - Tabular

Primer pair 1

	Sequence (5'->3')	Length	Tm	GC%	Self complementarity	Self 3' complementarity
**Forward primer**	GAAGATGGTATTCAAGCACGAA	22	56.79	40.91	4.00	4.00
**Reverse primer**	TTACAAACTCTCGGTGAAGG	20	55.04	45.00	3.00	0.00

Products on intended targets

Products on allowed targets

Products on allowed transcript variants

Products on potentially unintended templates

Products on target templates

>NC_008515.1 Escherichia phage RB32, complete genome

```
product length = 312
Forward primer  1       GAAGATGGTATTCAAGCACGAA  22
Template        60927   ......................  60906

Reverse primer  1       TTACAAACTCTCGGTGAAGG  20
Template        60616   ....................  60635
```

>NC_028780.1 Escherichia phage slur07, complete genome

```
product length = 312
Forward primer  1        GAAGATGGTATTCAAGCACGAA  22
Template        101790   ......................  101811

Reverse primer  1        TTACAAACTCTCGGTGAAGG  20
```

```
Template        102101  ....................  102082
```

>NC_031047.1 Escherichia phage HY03, complete genome

```
product length = 312
Forward primer  1      GAAGATGGTATTCAAGCACGAA  22
Template        62410  ......................  62431

Reverse primer  1      TTACAAACTCTCGGTGAAGG  20
Template        62721  ....................  62702
```

>NC_054919.1 Escherichia phage vB_EcoM_G4507, complete genome

```
product length = 312
Forward primer  1      GAAGATGGTATTCAAGCACGAA  22
Template        61794  ......................  61773

Reverse primer  1      TTACAAACTCTCGGTGAAGG  20
Template        61483  ....................  61502
```

>NC_054920.1 Escherichia phage vB_EcoM_G9062, complete genome

```
product length = 312
Forward primer  1      GAAGATGGTATTCAAGCACGAA  22
Template        62778  ......................  62757

Reverse primer  1      TTACAAACTCTCGGTGAAGG  20
Template        62467  ....................  62486
```

>NC_054922.1 Escherichia phage vB_EcoM_KAW1E185, complete genome

```
product length = 312
Forward primer  1      GAAGATGGTATTCAAGCACGAA  22
Template        61357  ......................  61336

Reverse primer  1      TTACAAACTCTCGGTGAAGG  20
Template        61046  ....................  61065
```

>NC_054934.1 Escherichia phage teqskov, complete genome

```
product length = 312
Forward primer  1       GAAGATGGTATTCAAGCACGAA  22
Template        122632  ......................  122653

Reverse primer  1       TTACAAACTCTCGGTGAAGG  20
Template        122943  ....................  122924
```

>MK327937.1 Escherichia phage vB_EcoM_G10400, complete genome

```
product length = 312
Forward primer  1      GAAGATGGTATTCAAGCACGAA  22
Template        61273  ......................  61252

Reverse primer  1      TTACAAACTCTCGGTGAAGG  20
Template        60962  ....................  60981
```

>MK327944.1 Escherichia phage vB_EcoM_G2540-3, complete genome

```
product length = 312
Forward primer  1       GAAGATGGTATTCAAGCACGAA  22
Template        61655  ......................  61634

Reverse primer  1       TTACAAACTCTCGGTGAAGG  20
Template        61344  ....................  61363
```

>MZ234013.1 Escherichia phage vB_EcoM-101112UKE3-1, complete genome

```
product length = 312
Forward primer  1       GAAGATGGTATTCAAGCACGAA  22
Template        160145  ......................  160166

Reverse primer  1       TTACAAACTCTCGGTGAAGG  20
Template        160456  ....................  160437
```

>MZ234027.1 Escherichia phage vB_EcoM-CHD2BS1, complete genome

```
product length = 312
Forward primer  1       GAAGATGGTATTCAAGCACGAA  22
Template        159167  ......................  159188

Reverse primer  1       TTACAAACTCTCGGTGAAGG  20
Template        159478  ....................  159459
```

>MZ234030.1 Escherichia phage vB_EcoM-CHD16UKE1, complete genome

```
product length = 312
Forward primer  1       GAAGATGGTATTCAAGCACGAA  22
Template        159133  ......................  159154

Reverse primer  1       TTACAAACTCTCGGTGAAGG  20
Template        159444  ....................  159425
```

>MZ234034.1 Escherichia phage vB_EcoM-G3F6, complete genome

```
product length = 312
Forward primer  1       GAAGATGGTATTCAAGCACGAA  22
Template        159114  ......................  159135

Reverse primer  1       TTACAAACTCTCGGTGAAGG  20
Template        159425  ....................  159406
```

>MZ234036.1 Escherichia phage vB_EcoM-G3F8, complete genome

```
product length = 312
Forward primer  1       GAAGATGGTATTCAAGCACGAA  22
Template        159114  ......................  159135

Reverse primer  1       TTACAAACTCTCGGTGAAGG  20
Template        159425  ....................  159406
```

>MZ234037.1 Escherichia phage vB_EcoM-G3F9, complete genome

```
product length = 312
Forward primer  1       GAAGATGGTATTCAAGCACGAA  22
Template        159114  ......................  159135

Reverse primer  1       TTACAAACTCTCGGTGAAGG  20
Template        159425  ....................  159406
```

>MZ234039.1 Escherichia phage vB_EcoM-G3G6, complete genome

```
product length = 312
Forward primer  1       GAAGATGGTATTCAAGCACGAA  22
Template        159112  ......................  159133

Reverse primer  1       TTACAAACTCTCGGTGAAGG  20
Template        159423  ....................  159404
```

>MZ234040.1 Escherichia phage vB_EcoM-G3G7, complete genome

```
product length = 312
Forward primer  1       GAAGATGGTATTCAAGCACGAA  22
Template        159239  ......................  159260

Reverse primer  1       TTACAAACTCTCGGTGAAGG  20
Template        159550  ....................  159531
```

>MZ234043.1 Escherichia phage vB_EcoM-PM123, complete genome

```
product length = 312
Forward primer  1       GAAGATGGTATTCAAGCACGAA  22
Template        160038  ......................  160059

Reverse primer  1       TTACAAACTCTCGGTGAAGG  20
Template        160349  ....................  160330
```

>MZ234053.1 Escherichia phage vB_EcoM-PM136, complete genome

```
product length = 312
Forward primer  1       GAAGATGGTATTCAAGCACGAA  22
Template        160109  ......................  160130

Reverse primer  1       TTACAAACTCTCGGTGAAGG  20
Template        160420  ....................  160401
```

>MZ436830.1 Escherichia phage JK1, complete genome

```
product length = 312
Forward primer  1      GAAGATGGTATTCAAGCACGAA  22
Template        60796  ......................  60775

Reverse primer  1      TTACAAACTCTCGGTGAAGG  20
Template        60485  ....................  60504
```

>OK272484.1 Escherichia phage JLBYU22, complete genome

```
product length = 312
Forward primer  1      GAAGATGGTATTCAAGCACGAA  22
```

```
Template        60666  ......................  60645

Reverse primer  1      TTACAAACTCTCGGTGAAGG  20
Template        60355  ....................  60374
```

>OR062944.1 Escherichia phage 303Ecol101PP, complete genome

```
product length = 312
Forward primer  1       GAAGATGGTATTCAAGCACGAA  22
Template        115181  ......................  115202

Reverse primer  1       TTACAAACTCTCGGTGAAGG  20
Template        115492  ....................  115473
```

>PP418987.1 Escherichia phage UPWr_E4, complete genome

```
product length = 312
Forward primer  1       GAAGATGGTATTCAAGCACGAA  22
Template        159579  ......................  159600

Reverse primer  1       TTACAAACTCTCGGTGAAGG  20
Template        159890  ....................  159871
```

>MH751506.1 Escherichia phage T2, complete genome

```
product length = 312
Forward primer  1      GAAGATGGTATTCAAGCACGAA  22
Template        61375  ......................  61354

Reverse primer  1      TTACAAACTCTCGGTGAAGG  20
Template        61064  ...............A....  61083
```

>NC_024125.2 Escherichia phage e11/2, complete genome

```
product length = 312
Forward primer  1      GAAGATGGTATTCAAGCACGAA  22
Template        63223  ......................  63202

Reverse primer  1      TTACAAACTCTCGGTGAAGG  20
Template        62912  ...............A....  62931
```

>NC_025449.1 Escherichia phage ECML-134, complete genome

```
product length = 312
Forward primer  1      GAAGATGGTATTCAAGCACGAA  22
Template        60536  ......................  60515

Reverse primer  1      TTACAAACTCTCGGTGAAGG  20
Template        60225  ...............A....  60244
```

>NC_054915.1 Escherichia phage vB_EcoM_G8, complete genome

```
product length = 312
Forward primer  1      GAAGATGGTATTCAAGCACGAA  22
Template        61411  ......................  61390

Reverse primer  1      TTACAAACTCTCGGTGAAGG  20
```

```
Template        61100  ...............A....  61119
```

>NC_054918.1 Escherichia phage vB_EcoM_G4498, complete genome

```
product length = 312
Forward primer  1      GAAGATGGTATTCAAGCACGAA  22
Template        61983  ......................  61962

Reverse primer  1      TTACAAACTCTCGGTGAAGG  20
Template        61672  ...............A....  61691
```

>NC_054926.1 Escherichia phage vB_EcoM_OE5505, complete genome

```
product length = 312
Forward primer  1      GAAGATGGTATTCAAGCACGAA  22
Template        62450  ......................  62429

Reverse primer  1      TTACAAACTCTCGGTGAAGG  20
Template        62139  ...............A....  62158
```

>NC_054933.1 Escherichia phage teqhad, complete genome

```
product length = 312
Forward primer  1      GAAGATGGTATTCAAGCACGAA  22
Template        67995  ......................  67974

Reverse primer  1      TTACAAACTCTCGGTGAAGG  20
Template        67684  ...............A....  67703
```

>MN022785.1 Escherichia phage VEc20, complete genome

```
product length = 312
Forward primer  1      GAAGATGGTATTCAAGCACGAA  22
Template        61544  ......................  61523

Reverse primer  1      TTACAAACTCTCGGTGAAGG  20
Template        61233  ...............A....  61252
```

>MN895435.1 Escherichia phage teqhal, complete genome

```
product length = 312
Forward primer  1       GAAGATGGTATTCAAGCACGAA  22
Template        145679  ......................  145700

Reverse primer  1       TTACAAACTCTCGGTGAAGG  20
Template        145990  ...............A....  145971
```

>MZ417521.1 Escherichia phage 132, complete genome

```
product length = 312
Forward primer  1      GAAGATGGTATTCAAGCACGAA  22
Template        61002  ......................  60981

Reverse primer  1      TTACAAACTCTCGGTGAAGG  20
Template        60691  ...............A....  60710
```

>OR757453.1 Escherichia phage BYEP01, complete genome

```
product length = 312
Forward primer  1      GAAGATGGTATTCAAGCACGAA  22
Template        17606  ......................  17627

Reverse primer  1      TTACAAACTCTCGGTGAAGG  20
Template        17917  ...............A....  17898
```

>NC_027983.1 Enterobacteria phage AR1 DNA, complete genome

```
product length = 312
Forward primer  1      GAAGATGGTATTCAAGCACGAA  22
Template        61077  ......................  61056

Reverse primer  1      TTACAAACTCTCGGTGAAGG  20
Template        60766  ............A..A....  60785
```

>NC_054917.1 Escherichia phage vB_EcoM_G50, complete genome

```
product length = 312
Forward primer  1      GAAGATGGTATTCAAGCACGAA  22
Template        61507  ......................  61486

Reverse primer  1      TTACAAACTCTCGGTGAAGG  20
Template        61196  .....G.........A....  61215
```

>NC_054921.1 Escherichia phage vB_EcoM_IME537, complete genome

```
product length = 312
Forward primer  1       GAAGATGGTATTCAAGCACGAA  22
Template        140565  ......................  140544

Reverse primer  1       TTACAAACTCTCGGTGAAGG  20
Template        140254  ............A..A....  140273
```

>NC_054928.1 Escherichia phage vB_vPM_PD112, complete genome

```
product length = 312
Forward primer  1       GAAGATGGTATTCAAGCACGAA  22
Template        134280  ......................  134259

Reverse primer  1       TTACAAACTCTCGGTGAAGG  20
Template        133969  ............A..A....  133988
```

>NC_054932.1 Escherichia phage teqdroes, complete genome

```
product length = 312
Forward primer  1       GAAGATGGTATTCAAGCACGAA  22
Template        159992  ......................  160013

Reverse primer  1       TTACAAACTCTCGGTGAAGG  20
Template        160303  ............A..A....  160284
```

>MK327928.1 Escherichia phage vB_EcoM_G2133, complete genome

```
product length = 312
Forward primer  1      GAAGATGGTATTCAAGCACGAA  22
Template        61971  ......................  61950

Reverse primer  1      TTACAAACTCTCGGTGAAGG  20
Template        61660  ............A..A....  61679
```

>MK977694.1 Escherichia phage vB_EcoM-Sa45lw, complete genome

```
product length = 312
Forward primer  1      GAAGATGGTATTCAAGCACGAA  22
Template        60919  ......................  60898

Reverse primer  1      TTACAAACTCTCGGTGAAGG  20
Template        60608  ............A..A....  60627
```

>MT968995.1 Escherichia phage vB_EcoM_WL-3, complete genome

```
product length = 312
Forward primer  1      GAAGATGGTATTCAAGCACGAA  22
Template        59609  ......................  59588

Reverse primer  1      TTACAAACTCTCGGTGAAGG  20
Template        59298  ............A..A....  59317
```

>NC_019505.1 Escherichia phage wV7, complete genome

```
product length = 312
Forward primer  1      GAAGATGGTATTCAAGCACGAA  22
Template        61399  ......................  61378

Reverse primer  1      TTACAAACTCTCGGTGAAGG  20
Template        61088  ............A..A....  61107
```

>NC_028448.1 Escherichia phage slur14, complete genome

```
product length = 312
Forward primer  1     GAAGATGGTATTCAAGCACGAA  22
Template        4117  ......................  4096

Reverse primer  1     TTACAAACTCTCGGTGAAGG  20
Template        3806  ............A..A....  3825
```

>OL770073.2 Escherichia phage vB_EcoM-RPN226, complete genome

```
product length = 312
Forward primer  1       GAAGATGGTATTCAAGCACGAA  22
Template        144142  .....C................  144163

Reverse primer  1       TTACAAACTCTCGGTGAAGG  20
Template        144453  ...............A....  144434
```

>NC_000866.4 Enterobacteria phage T4, complete genome

```
product length = 312
Forward primer  1      GAAGATGGTATTCAAGCACGAA  22
```

```
Template        63868  ......................  63847

Reverse primer  1      TTACAAACTCTCGGTGAAGG  20
Template        63557  .........T..A..A....  63576
```

>NC_027349.1 Escherichia phage HY01, complete genome

```
product length = 312
Forward primer  1      GAAGATGGTATTCAAGCACGAA  22
Template        61009  .....C................  60988

Reverse primer  1      TTACAAACTCTCGGTGAAGG  20
Template        60698  ............A..A....  60717
```

>OR062946.1 Escherichia phage 310Ecol104PP, complete genome

```
product length = 312
Forward primer  1      GAAGATGGTATTCAAGCACGAA  22
Template        45916  .....C................  45895

Reverse primer  1      TTACAAACTCTCGGTGAAGG  20
Template        45605  .....G.........A....  45624
```

>OR757454.1 Escherichia phage BYEP02, complete genome

```
product length = 312
Forward primer  1       GAAGATGGTATTCAAGCACGAA  22
Template        101640  .....C................  101661

Reverse primer  1       TTACAAACTCTCGGTGAAGG  20
Template        101951  ............A..A....  101932
```

>KR233165.1 Escherichia phage PEC04, complete genome

```
product length = 312
Forward primer  1     GAAGATGGTATTCAAGCACGAA  22
Template        8286  .....C................  8307

Reverse primer  1     TTACAAACTCTCGGTGAAGG  20
Template        8597  ............A..A....  8578
```

If you want to allow any of the unintended targets, check the box(es) next to the ones you accept and try again to re-search for specific primers Submit

Help

FOLLOW NCBI

Connect with NLM
